# Comparative Genomics Reveal That Host-Innate Immune Responses Influence the Clinical Prevalence of *Legionella pneumophila* Serogroups

**DOI:** 10.1371/journal.pone.0067298

**Published:** 2013-06-27

**Authors:** Mohammad Adil Khan, Natalie Knox, Akriti Prashar, David Alexander, Mena Abdel-Nour, Carla Duncan, Patrick Tang, Hajera Amatullah, Claudia C. Dos Santos, Nathalie Tijet, Donald E. Low, Christine Pourcel, Gary Van Domselaar, Mauricio Terebiznik, Alexander W. Ensminger, Cyril Guyard

**Affiliations:** 1 Public Health Ontario, Toronto, Ontario, Canada; 2 Department of Laboratory Medicine and Pathobiology, Faculty of Medicine, University of Toronto, Toronto, Ontario, Canada; 3 National Microbiology Laboratory, Public Health Agency of Canada, Winnipeg, Manitoba, Canada; 4 Cell and Systems Biology and Biological Sciences, University of Toronto at Scarborough, Scarborough, Ontario, Canada; 5 The Keenan Research Centre of the Li Ka Shing Knowledge Institute of St. Michael's Hospital, Toronto, Ontario, Canada; 6 Mount Sinai Hospital, Toronto, Ontario, Canada; 7 Institut de Génétique et Microbiologie, Université Paris-Sud, Paris, France; 8 Department of Molecular Genetics, Faculty of Medicine, University of Toronto, Toronto, Ontario, Canada; University of Louisville, United States of America

## Abstract

*Legionella pneumophila* is the primary etiologic agent of legionellosis, a potentially fatal respiratory illness. Amongst the sixteen described *L. pneumophila* serogroups, a majority of the clinical infections diagnosed using standard methods are serogroup 1 (Sg1). This high clinical prevalence of Sg1 is hypothesized to be linked to environmental specific advantages and/or to increased virulence of strains belonging to Sg1. The genetic determinants for this prevalence remain unknown primarily due to the limited genomic information available for non-Sg1 clinical strains. Through a systematic attempt to culture *Legionella* from patient respiratory samples, we have previously reported that 34% of all culture confirmed legionellosis cases in Ontario (n = 351) are caused by non-Sg1 *Legionella*. Phylogenetic analysis combining multiple-locus variable number tandem repeat analysis and sequence based typing profiles of all non-Sg1 identified that *L. pneumophila* clinical strains (n = 73) belonging to the two most prevalent molecular types were Sg6. We conducted whole genome sequencing of two strains representative of these sequence types and one distant neighbour. Comparative genomics of the three *L. pneumophila* Sg6 genomes reported here with published *L. pneumophila* serogroup 1 genomes identified genetic differences in the O-antigen biosynthetic cluster. Comparative optical mapping analysis between Sg6 and Sg1 further corroborated this finding. We confirmed an altered O-antigen profile of Sg6, and tested its possible effects on growth and replication in *in vitro* biological models and experimental murine infections. Our data indicates that while clinical Sg1 might not be better suited than Sg6 in colonizing environmental niches, increased bloodstream dissemination through resistance to the alternative pathway of complement mediated killing in the human host may explain its higher prevalence.

## Introduction

Legionellosis is a potentially fatal infectious disease caused by Gram-negative, aerobic bacteria belonging to the genus *Legionella* [1], [2]. Among 54 known *Legionella* species, *L. pneumophila* is the major cause of outbreaks (91.5%), and was the etiological agent of the first recognized outbreak in 1976 during a convention of the American Legion in Philadelphia [3], [4], [5], [6]. The severity of this disease ranges from a mild respiratory illness to a rapidly fatal pneumonia [1], [7]. The case fatality rate of legionellosis is between 40–80% in untreated immuno-suppressed patients but can be reduced to 5–30% with appropriate case management [7]. Legionellosis is a major public health concern in industrialized nations [8], [9], [10]. From 2000 to 2009, a 217% increase in legionellosis cases was reported in the United States [11], [12].

*Legionella spp.* are found worldwide and can be detected in up to 80% of man-made freshwater sites [13], [14], [15], [16], [17], [18], [19]. More recently, a study showed that *Legionella* spp can persistently colonize aquifers over several years [19]. In natural and in man-made water systems, *L. pneumophila* may exist as planktonic cells or as biofilms [20], [21]. The bacteria can also be isolated from different protozoa in the environment [22], [23], [24]. This is an essential step in *L. pneumophila*’s life cycle, since the bacteria are able to replicate in the environment within the protozoa host [23], [25]. The human infection results from the inhalation of aerosols contaminated with either infected protozoa or free floating *L. pneumophila* [26], [27]. The general model predicts, that upon inhalation, the infection starts with the invasion and replication in macrophage and epithelial cells of the lung [28], [29]. Once inside the cell, *Legionella* replicate with in a non-acidified vacuolar environment [30], [31]. While many genes required for surviving in amoeba are yet to be described, some like the *dot/icm* genes [32], [33] have shown to be essential for survival in both the amoeba and the human host.

*L. pneumophila* have been classified into sixteen serogroups based on reactivity to specific monoclonal antibodies [34], [35]. While these serogroups have been reported worldwide, Sg1 has historically been identified in most clinical cases (84.2%) [6]. The most widely used clinical diagnostic method for all suspected legionellosis cases are rapid, non-invasive, urine detection tests that recognize the presence of soluble antigen [36]. These clinical tests provide high sensitivity for Sg1 infection diagnosis but they are unable to detect most non-Sg1 strains [37], [38]. Recently, we reported that 34% of culture confirmed cases of legionellosis in Ontario (n = 351) were caused by non-Sg1 *L. pneumophila* species in the last 3 decades [39]. Interestingly such a high percentage of non-Sg1 was also reported in some Scandinavian countries [40]. In a comparative clinical and environmental distribution analysis study, Sg1 was shown to constitute 28.2% of the *Legionella* spp. bacteria isolated from man-made water systems (n = 2,747) but 95.4% of clinical samples (n = 259) [41]. This suggests that the clinical prevalence of Sg1 might be independent of predominance in the environment, but rather to fitness or virulence advantages in the human host.

The underlying reasons for the clinical prevalence of Sg1 strains are a subject of active debate. A large, microarray multigenome analysis of 217 *L. pneumophila* isolates previously suggested that the clinical predominance of Sg1 may be linked to its specific lipopolysaccharide [42]. In this study, thorough population-based molecular epidemiology, we first identified the most clinically prevalent Sg6 strain among non-Sg1 clinical isolates in Ontario. Next, by combining next-generation sequencing with *in vitro* and *in vivo* experimental models, we performed a comparative analysis with the closest identified phylogenetic Sg1 (strain Philadelphia). Our results support a model in which increased resistance to serum complement, rather than variation in environmental fitness, explains the relative clinical prevalence of Sg1.

## Results

### Identification of Phylogenetic Clusters within non-Sg1 *L. pneumophila* Clinical Isolates

**Figure 1:**
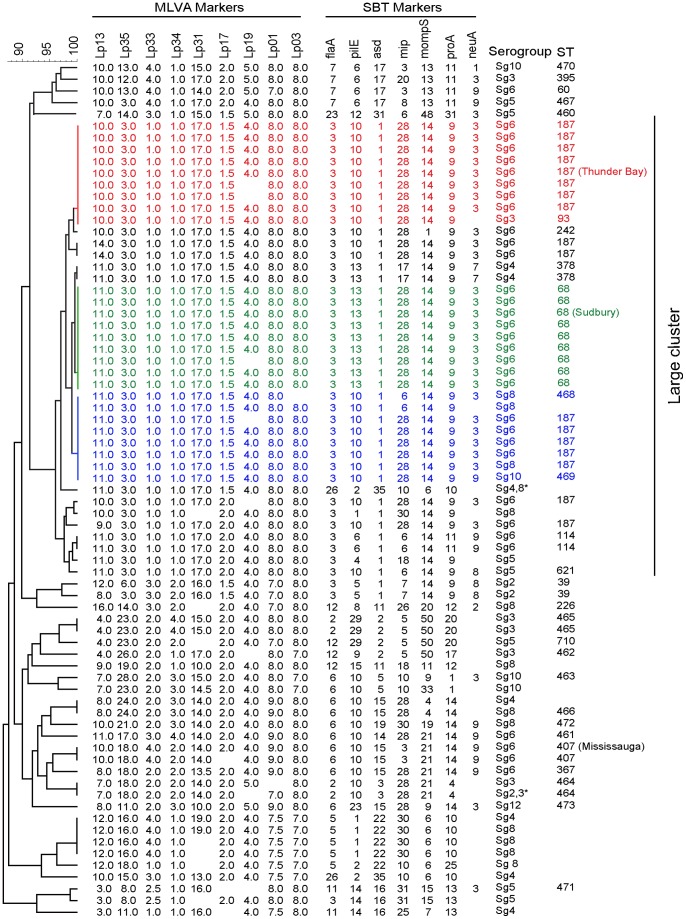
Hybrid SBT-MLVA typing of a population based clinical repository of *L. pneumophila*. Phylogenetic clusters were formed by UPGMA analysis of the combined typing data. Cluster 1 is identified in blue, while cluster 2 and 3 are shown in green and red, respectively.

To identify prevalent population based clusters of non-Sg1 *L. pneumophila* clinical strains in Ontario, we conducted a phylogenetic analysis on 73 clinical isolates recovered between 1980 and 2009 using sequence based typing (SBT) and multiple-locus variable number tandem repeat analysis (MLVA). SBT based phylogenetic comparisons between different strains is accomplished by observing sequence variations of 7 allelic markers including house-keeping genes and known virulence factors [43], [44]. MLVA takes advantage of the polymorphisms of 9 tandemly repeated DNA sequences [45]. The UPGMA analysis based on a matrix of pairwise allelic differences between all the sequence types (ST) of our dataset identified a large cluster (n = 40) comprising most of the Sg6 isolates of our repository. Isolates of this large cluster showed less phylogenetic diversity compared with other clusters and it encompassed 2 two main subclusters (Figure S1). The phylogenetic subcluster 1 included 17 isolates of Sg6 and ST 187 (Figure S1), while the phylogenetic subcluster 2 included 9 isolates who were all identified as Sg6 ST 68 (Figure S1). MLVA typing of this dataset, identified a large phylogenetic cluster as well, comprising two subclusters, where cluster 1 grouped 20 isolates, and included Sg6 ST 187 and Sg6 ST 68, along with Sg4, Sg8 and Sg10 strains (Figure S2). Interestingly, cluster 2 had 10 isolates, out of which 8 were Sg6 ST 187, 1 was a Sg6 ST242 and 1 was a Sg3 ST93 (Figure S2). To further increase the discriminatory power of the molecular typing methods [46], we next constructed a phylogenetic tree combining SBT and MLVA profiles (Figure 1). This combined analysis confirmed that the largest phylogenetic cluster of non-sg1 clinical isolates comprised mainly of Sg6 ST187 and Sg6 ST68. In contrast to several other molecular types such Sg6 ST407 which had only been isolated over a period of 5 years, Sg6 ST187 and Sg6 ST68 strains seemed clonally stable, since isolates with identical sequence types have been obtained for 25 to 30 year periods of our repository (initiated in 1980). For the purposes of further analysis we selected strain Thunder Bay, Sudbury and Mississauga isolates as the representative ST187, ST68 and ST407, respectively. The high-prevalence of Sg6 among non-Sg1s was also confirmed through an inspection of the European Working Group for *Legionella* Infections (EWGLI) database (http://www.ewgli.org/) and has been reported independently elsewhere [41].

### The Optical Map of *L. pneumophila* Sg6 str. Thunder Bay and Sg12 str. 570-CO-H are Identical

**Figure 2:**
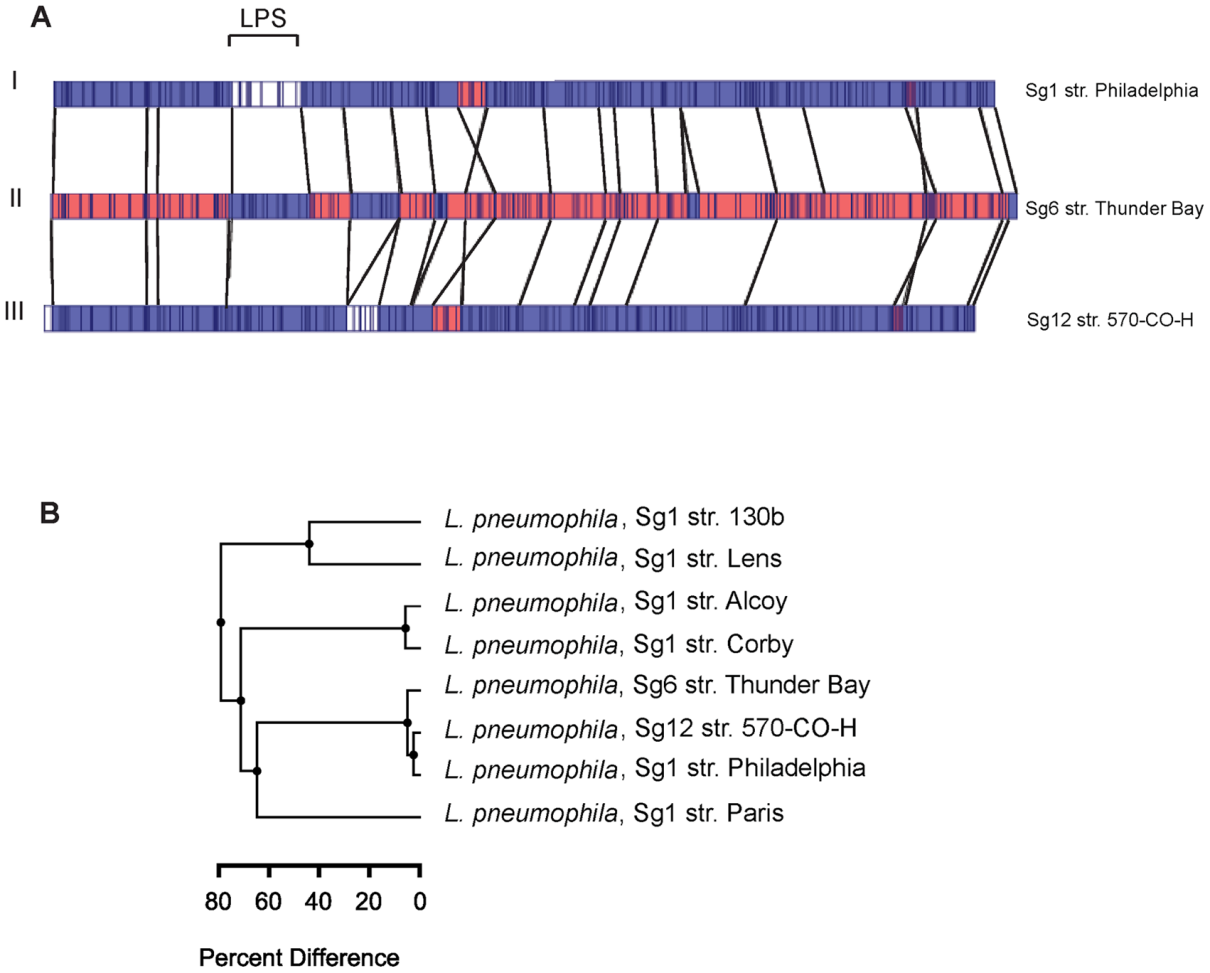
Optical Maps and genome based comparisons of *L. pneumophila* strains. (A) Optical map of *L. pneumophilla* Sg6 str. Thunder Bay (Middle) compared to Sg1 strains Philadelphia (Top) and Sg12 570-CO-H (Bottom). The regions in white indicate unique gene clusters, while areas in blue depict high similarity to Sg6. Regions in red are conserved between all three genome sequences. (B) UPGMA based cluster analysis of optical maps of sequenced *L. pneumophila*.

Optical mapping is an emerging technology used for accurate genome assemblies [47] and for conducting comparative chromosomal analysis [48]. It allows for rapid identification of divergent regions, chromosomal rearrangement, and inversions [48], [49], [50]. We compared the relatedness of Sg6 str. Thunder Bay with other non-Sg1 and Sg1 strains, whose genomic sequences are available. First, we constructed an optical map of Sg6 str. Thunder Bay and compared it to *in silico* maps of Sg1 str. Philadelphia and Sg12 str. 570-CO-H (Figure 2A). The optical maps of Sg6 str. Thunder Bay and Sg1 str. Philadelphia only showed divergence in a 40 kB segment. This segment corresponded to genes involved in O-antigen biosynthesis. In contrast, Sg12 str. 570-CO-H showed a similar restriction pattern with Sg6 str. Thunder Bay. Secondly, we constructed an evolutionary tree based on optical maps of Sg6 str. Thunder Bay, Sg12 str. 570-CO-H and all sequenced and fully assembled Sg1 strains (Figure 2B). This analysis confirmed that the clinical non-Sg1 strains, Sg6 str. Thunder Bay and Sg12 str. 570-CO-H share a high degree of homology, and are phylogenetic neighbours of Sg1 str. Philadelphia.

### *L. pneumophila* Sg6 str. Thunder Bay Generates Higher Amounts of Biofilm Compared to Sg1 str. Philadelphia

**Figure 3:**
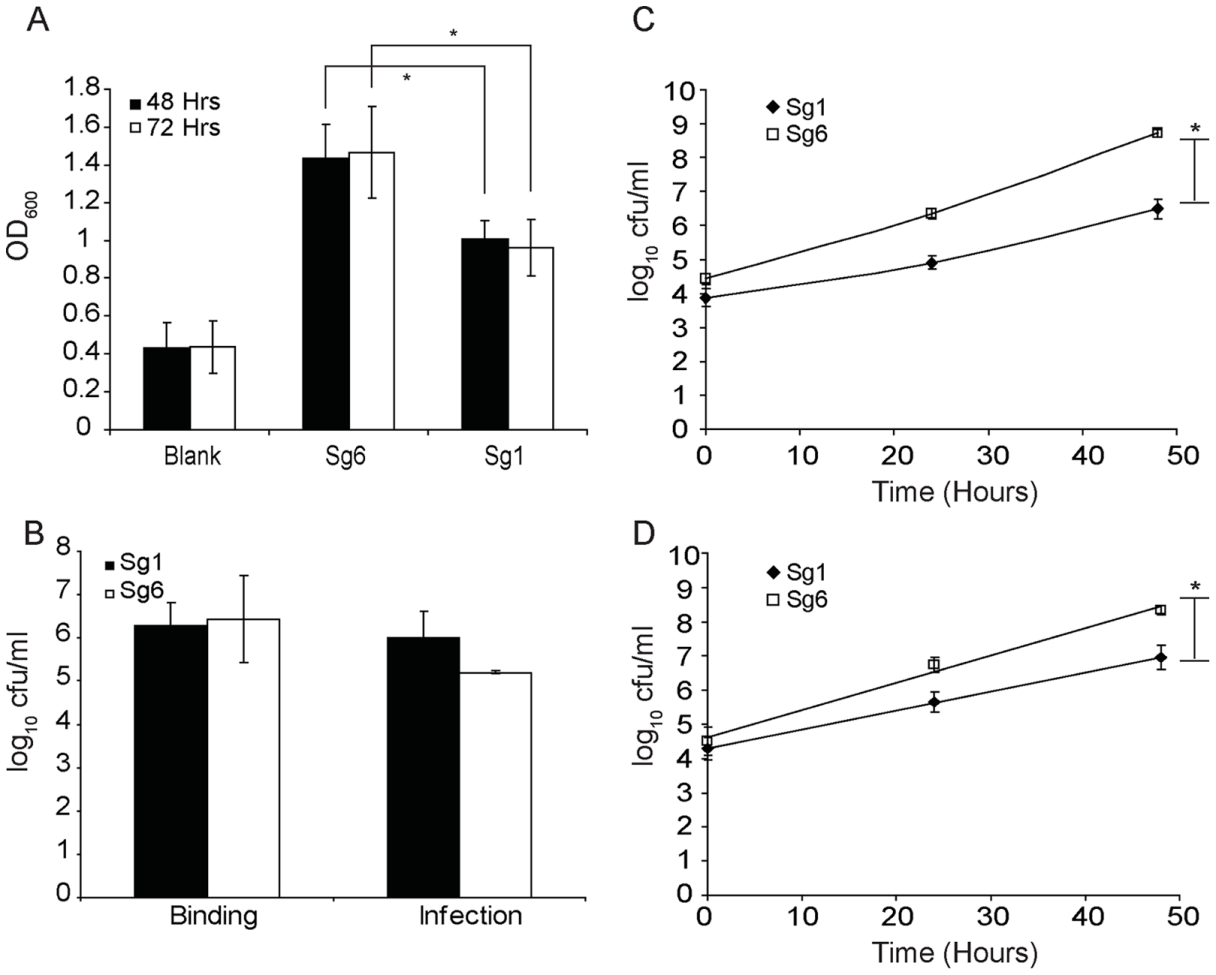
Intracellular growth of *L. pneumophila*. (A) Biofilm production by Sg1 and Sg6 (crystal violet staining, OD_600 nm_). (B) Binding and invasion of Sg6 str. Thunder Bay compared to Sg1 str. Philadelphia to/within human NCI-H292 lung epithelial cells. (C) Intracellular replication of *L. pneumophilla* in *A. castellani*. The magnitude of replication is reported in log10 CFU/ml. (D) Intracellular growth of Sg6 str. Thunder Bay (square) compared to Sg1 str. Philadelphia (diamond) within U937 derived Human Macrophage cells. Each data point is an average of three independent experiments. For each experiment data was collected from 3 wells and a mean value was determined. *denotes statistical significance as determined by a two-tailed student’s t-test with a P-value <0.05.

The comparative optical map analyses identified possible serogroup specific genomic parameters. In order to investigate if these differences result in possible serogroup specific advantages between Sg1 str. Philadelphia and Sg6 str. Thunder Bay, we conducted comparative biological analysis at different stages of the *L. pneumophila* life-cycle. A strategy used by *L. pneumophila* to survive in anthropogenic aqueous environment is to generate biofilms [51]. The ability to generate higher amounts of biofilm can be directly correlated with persistence in the environment [52]. We determined that Sg6 str. Thunder Bay produces significantly higher levels of biofilm compared to Sg1 str. Philadelphia by 48 hours (P<0.05; Figure 3A). Furthermore, this difference further increased by 72 hours (P<0.05). This suggests that while Sg1 are able to generate significant amounts of biofilm, the Sg6 might possess an advantage that could explain its higher reported prevalence in some environmental surveys [53].

### *L. pneumophila* Sg6 str. Thunder Bay Replicates more Efficiently than Sg1 str. Philadelphia within *A. castellani* Amoebae and U937 Derived Macrophages, but not in Lung Epithelial Cells

In order to determine a possible advantage that either Sg1 or Sg6 may possess in infecting humans and surviving in the environment, we compared the replication rates of Sg1 str. Philadelphia and Sg6 str. Thunder Bay over 48 hours in the human lung epithelial cell line H292, U937 macrophages and *Acanthamoeba castellani* amoeba models [54], [55], [56]. While no differences were observed in the lung epithelial cells (Figure 3B), significant increase of Sg6 str. Thunder Bay counts over Sg1 str. Philadelphia was seen by 48 hours in the macrophage model (Figure 3C). Similarly in amoeba, while no discernable difference was observed between the Sg1 and Sg6 strains one hour after uptake, by 24 hours, counts for the Sg6 strain were 28-fold higher compared to Sg1 str. Philadelphia (Figure 3D). At 48 hours, this difference had increased to 168-fold, suggesting that Sg6 str. Thunder Bay is able to infect and replicate more effectively than Sg1 str. Philadelphia in *A. castellani* model. Taken together, these results suggest that the Sg6 str. Thunder Bay replicates more efficiently than Sg1 str. Philadelphia within amoebae and macrophages. Thus Sg6 str. Thunder Bay might possess a fitness advantage in the environmental niche over the Sg1 str. Philadelphia, which is an unlikely explanation for the observed differences in clinical prevalence.

### The most Prevalent Molecular Types of Sg6 Strains Share a High Degree of Genomic Similarity

**Figure 4:**
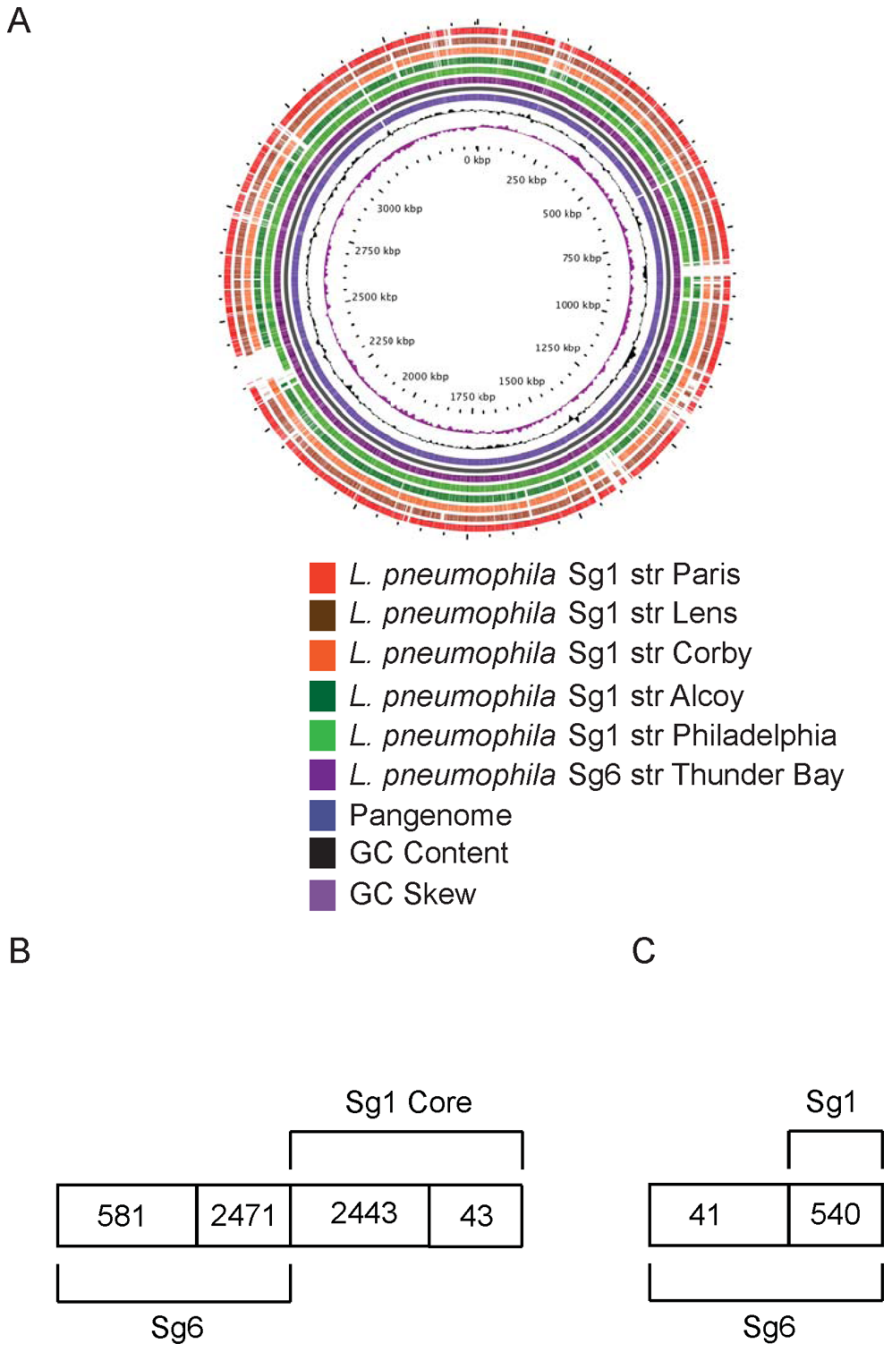
Pangenome comparison of *L. pneumophila*. (A) BLAST map of *L. pneumophila* Sg6 str. Thunder Bay against several Sg1 strains. (B) Number of conserved (>65% identity) and divergent (<65% identity) proteins between Sg6 and a subset of core **5**proteins of Sg1. Numbers showed in italics represent number of proteins that were considered homologous. (C) Conserved (>65% identity) and divergent (<65% identity) proteins among the unique Sg6 genes from panel B and all known Sg1 genes.

To identify the factors that may be contributing to the population structure of *L. pneumophila* in clinical settings, we performed whole genome sequencing of Sg6 str. Thunder Bay. The *L. pneumophila* Sg6 str. Thunder Bay (accession number: CP003730) genome consists of a 3455167-bp chromosome with an average GC content of 38.24% (Table S1). A total of 3052 protein-encoding genes are predicted, 2299 (75.3%) of which have been assigned a putative function. A BLAST map analysis of Thunder Bay against five reference Sg1 strains showed several conserved regions (Figure 4A). Furthermore, high genomic strand asymmetry identified through the GC skew suggested no abnormalities in the assembly of the genome [57] (Figure 4A). To determine plasticity of the Sg6 genomes, we selected two additional strains from our clinical repository, Sg6 str. Sudbury (Accession number PRJNA170020) and Sg6 str. Mississauga (Accession number PRJNA170014), and sequenced their genome using Illumina technology (paired-end). The genomes were *de novo* assembled into 29 and 37 contigs, respectively. By including these strains in our analysis, we report genomic data on most sequence types that were identified as Sg6 in our database. The genome size of Sg6 str. Sudbury (3351893 bp) and Sg6 str. Mississauga (3384301 bp) were comparable to that of the Sg6 str. Thunder Bay. Reference assembly of raw Illumina reads to the finished Sg6 str. Thunder Bay genome also indicated that Sg6 str. Sudbury was more closely related to Sg6 str. Thunder Bay (∼4800 SNPs) than Sg6 str. Mississauga (∼79,000 SNPs) (data not shown). Taking these SNP variations into consideration, the overall polymorphism between Sg6 str. Thunder Bay and Sg6 str. Sudbury only represents 0.13% of the Sg6 str. Thunder Bay genome while the polymorphism between Sg6 str. Mississauga and Sg6 str. Thunder Bay represents 2.28% of Sg6 str. Thunder Bay genome.

**Figure 5:**
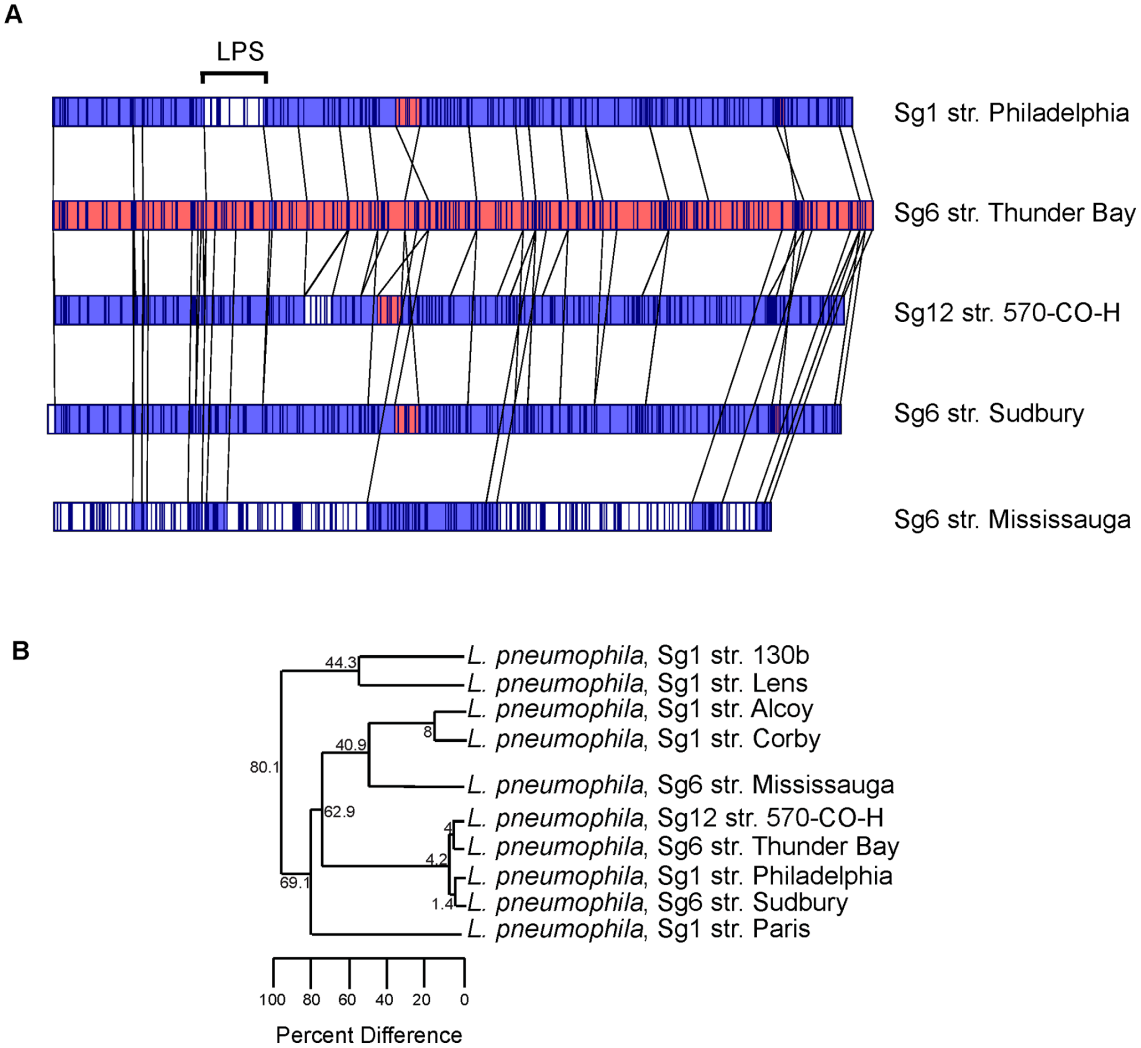
Optical Maps and genome based comparisons of *L. pneumophila* strains. (A) Optical map of *L. pneumophilla* Sg6 str. Thunder Bay (Middle) compared to Sg1 strains Philadelphia (Top) and Sg12 570-CO-H (Bottom). The regions in white indicate unique gene clusters, while areas in blue depict high similarity to Sg6. Regions in red are conserved between all three genome sequences. (B) UPGMA based cluster analysis of optical maps of sequenced *L. pneumophila*. Percent difference at each node is indicated.

The *in silico* optical maps deducted from concatenated whole genome sequencing contigs of Sg6 str. Sudbury and Sg6 str. Mississauga were next compared to the optical maps of Sg6 str. Thunder Bay, Sg1 str. Philadelphia and Sg12 str. 570-CO-H (Figure 5A). Similarly to Sg12 str. 570-CO-H and Sg6 str. Thunder Bay, Sg6 str. Sudbury showed high similarity with Sg1 str. Philadelphia with the exception of the O-antigen biosynthesis segment. In contrast, differences between optical maps of Sg6 str. Mississauga and other strains were distributed around the chromosome whereas the O-antigen biosynthesis segment was only partially conserved. An evolutionary tree based on optical maps of all sequenced Sg6 strains of our study, Sg12 str. 570-CO-H and all sequenced and fully assembled Sg1 strains was next constructed (Figure 5B). This analysis identified a cluster of strains with a relative low diversity compared to other genomes, this cluster included Sg6 str. Thunder Bay, Sg6 str. Sudbury, and Sg12 str. 570-CO-H and Sg1 str. Philadelphia. In contrast, the low prevalent Sg6 str. Mississauga did not cluster with Sg6 str. Thunder Bay and was relatively distant from its closest phylogenetic neighbours including Sg1 str. Alcoy and Sg1 str. Corby.

### Sg6 Genes Involved in O-antigen Biosynthesis Share Little Homology to the Sg1 Genomes

In order to identify genes that might confer differential prevalence of *L. pneumophila* serogroups, we compared the 2486 Sg1 proteins that have been previously described as the Sg1 core [58] for amino acid sequence homology with the Sg6 str. Thunder Bay predicted proteins. Forty three Sg1 core proteins shared less than 65% homology and were defined as divergent (Table S2, Figure 4B). While 35 amongst these were described as hypothetical proteins (HP), there was a cluster (∼40 kB) of 11 predicted proteins that corresponded to O-antigen biosynthesis (lpg0738 to lpg0786). This result is in agreement with the optical map based comparative analysis between Sg1 str. Philadelphia and Sg6 str. Thunder Bay, which identified the O-antigen biosynthetic cluster of Sg1 as divergent. This is also in agreement with a previous study revealing that Sg1 LPS gene cluster spaning lpp0827 (*galE*) to lpp0843 of *L. pneumophila* Sg1 str. Paris is highly divergent from those of Sg6, Sg10, Sg12, Sg13 and Sg14 [59]. Some other predicted proteins that were outside of this cluster included MreD (lpg0813) and LvrB (lpg1258). The role of LvrB has been characterized in several bacterial pathogens and has been shown to be essential in the formation of the Type IVA (lvh) secretion system [60], [61], [62], [63]. MreD has also been described in several bacteria, and has been shown to be involved in controlling cell wall biosynthesis and bacterial morphology [64], [65]. In our study, no morphological difference was observed between the Sg1 and Sg6 using differential interference contrast microscopy (data not shown).

In a reverse comparison, 581 Sg6 str. Thunder Bay proteins were defined as divergent compared to the Sg1 core. Amongst these only 41 were considered divergent to all *L. pneumophila* in the Genbank database (Table 1, Figure 4C). Twenty three of these Sg6-specific proteins were identified from the O-antigen biosynthetic cluster, and included proteins like Wzm (lp6_749), Wzt (lp6_750), GalE (lp6_758) and WecA (lp6_759), whose role in O-antigen biosynthesis is well described [66], [67], [68], [69]. Notably a previous study failed to identify the presence of many of the genes coding for O – antigen biosynthesis proteins by DNA microarray in all non-Sg1 tested [42]. Our results suggest that this may have been due to low sequence homology of these genes with their Sg1 orthologs. Interestingly, the Thunder Bay O-antigen cluster was fully conserved in the Sg6 str. Sudbury, but showed low homology in Wzt and *N*-actyltransferase when compared to Sg6 str. Mississauga.

**Table 1:** Divergent proteins present in Sg6 str. Thunder Bay compared to all sequenced Sg1 genomes in the Genbank database.

Sg6 Locus	Gene Name	Gene Description	Identityto Sg1	Sg1Locus	GeneName	Gene Description
Lp6_186		HP	43	lpg0112		HP
Lp6_749	Wzm	LPS O-antigen ABC transporter	48	lpp0837	Wzm	LPS O-antigen ABC transporter
Lp6_750	Wzt	LPS O-antigen ABC transporter	63	lpg0773	Wzt	LPS O-antigen ABC transporter
Lp6_755		HP	52	lpg0763		HP
Lp6_758	GalE	UDP-glucose 4-epimerase	61	lpg0761		HP
Lp6_759	WecA	α-*N*-acetylglucosaminyltransferase	57	lpg0762	WecA	O-antigen initiating glycosyl transferase
Lp6_761		HP	48	lpg0788		HP
Lp6_762		GCN5-related *N*-acetyltransferase	29	lpp1089		Streptomycin 3''-adenylyltransferase
Lp6_763	PseG	Pseudaminic acid biosynthesis-associated protein				
Lp6_764		Glutamate-1-semialdehyde 2,1-aminomutase	37	lpg0467	LasB	Zinc metalloprotease
Lp6_765	GlmU1	Acylneuraminate cytidylyltransferase	31	lpg1919	KdsB	3-deoxy-manno-octulosonate cytidylyltransferase
Lp6_766		Acetyltransferase	50	lpg2848		
Lp6_767	HisF4	Imidazoleglycerol-phosphate synthase	35	lpg0749	HisF	Imidazole glycerol phosphate synthase
Lp6_768	HisH2	Imidazole glycerol phosphate synthase	48	lpp2859	HisH	Imidazole glycerol phosphate synthase
Lp6_769		LPS biosynthesis protein	38	lpg0786		HP
Lp6_770		Aryl-alcohol dehydrogenase-like oxidoreductase	24	lpg2848		Ribonuclease
Lp6_771	DegT	Aminotransferase	52	lpl0206		HP
Lp6_772	CapD	Polysaccharide biosynthesis protein	59	lpg0561	PhaB	Acetoacetyl-CoA reductase
Lp6_773		HP	38	lpg0635		
Lp6_774		Capsule polysaccharide biosynthesis protein				
Lp6_775	NeuB	*N*-acetylneuraminic acid synthetase	38	lpp0818	NeuB	*N*-acetylneuraminic acid synthetase
Lp6_776		HP	47	lpa3427		HP
Lp6_777		HP				
Lp6_780		HP	31	lpg0774		HP
Lp6_781		Dehydrogenase	29	lpg1888		HP
Lp6_782		Dehydrogenase	36	lpg2974	Psd	Phosphatidylserine decarboxylase
Lp6_825		HP	47	lpg1344	DedE	Colicin V
Lp6_828		UDP-*N*-acetyl-D-galactosamine dehydrogenase	23	lpg1942		3-hydroxyacyl CoA dehydrogenase
Lp6_829		UDP-glucose 4-epimerase	33	lpg2214		Nucleoside-diphosphate sugar epimerase
Lp6_830		Starch synthase	35	lpp3018		HP
Lp6_831		HP	22	lpg2485		HP
Lp6_832		HP	32	lpg2015	ProC	Pyrroline-5-carboxylate reductase
Lp6_833		Glycosyl transferase family 2	35	lpg1183		HP
Lp6_976		HP	56	lpg0981		HP
Lp6_977	YwfO	Phosphohydrolase	45	lpg1267		HP
Lp6_1186		HP				
Lp6_1249	XerD	Integrase/recombinase				
Lp6_2002		integrase	59	lpc1833		HP
Lp6_2003		Protein of unknown function DUF1016	34	lpg1228		HP
Lp6_2041		HP	50	lpp0850		HP
Lp6_2164		HP	44	lpg1480	MutH	DNA mismatch repair protein

Proteins were defined as divergent when they shared less than 65% homology and 75% coverage. HP identifies hypothetical proteins.

Four Sg6 str. Thunder Bay proteins were defined as unique based on sequence homology with predicted proteins from all Sg1 genomic sequences (Table 1). Amongst these, three predicted proteins are coded by genes laying outside the O-antigen cluster and were classified as hypothetical proteins and XerD (lp6_1224), a site specific recombinase (Blakely and Sherratt, 1994). The remaining two proteins are coded by genes situated within the O-antigen cluster and included PseG (lp6_763), whose homologs have been shown to play a role in pseudaminic acid biosynthesis in *Campylobacter jejuni* and *Pseudomonas aeruginosa* [70], [71]. Even though pseudaminic acid has not been detected in *L. pneumophila*, a nine-carbon alpha-keto acid called legionaminic acid which belongs to the sialic acid family is generated by NeuA, NeuB and NeuC (Glaze et al., 2008). This acid is known to be a structural component of the *L. pneumophila* O – antigen. In agreement with a previous comparative analysis of *L. pneumophila* serogroup LPS clusters [59], this divergent O – antigen biosynthesis cluster were 100% homologous with corresponding *L. pneumophila* Sg12 str. 570-CO-H proteins (Table 2). At the DNA level, the region spanning Lp6_758 (*galE*) to Lp6_782 of Sg6 str. Thunder Bay showed respectively a 99% and 100% identity with the previously reported regions of Sg6 str. ATCC33215 and Sg12 str. ATCC43290 [59].

**Table 2:** Comparative proteomics analysis between divergent proteins identified in Table 1 and *L. pneumophila* Sg12 str. 570-CO-H.

Sg6 Locus	GeneName	Gene Description	Identity to Sg12	Sg12 Locus	Gene Name	Gene Description
Lp6_186		HP	82		lp12_2703	Phenylalanyl tRNA synthetase
Lp6_749	Wzm	LPS O-antigen ABC transporter	100	Wzm	lp12_0766	Polysaccharide ABC transporter
Lp6_750	Wzt	LPS O-antigen ABC transporter	100	Wzt	Lp12_0767	LPS O-antigen ABC transporter
Lp6_755		HP	100		lp12_0772	HP
Lp6_758	GalE	UDP-glucose 4-epimerase	100		lp12_0775	Putative NAD dependent epimerase
Lp6_759	WecA	α-*N*-acetylglucosaminyltransferase	100		lp12_0776	α-*N-* acetylglucosaminyltransferase
Lp6_761		Hypothetical protein	100		lp12_0778	HP
Lp6_762		GCN5-related *N*-acetyltransferase	100		lp12_0779	*N*-Acyltransferase
Lp6_763	PseG	Pseudaminic acid biosynthesis-associated protein	100		lp12_0780	putative polysaccharide biosynthesis protein
Lp6_764		Glutamate-1-semialdehyde 2,1-aminomutase	100		lp12_0781	Putative aminotransferase class-III
Lp6_765	GlmU1	Acylneuraminate cytidylyltransferase	100		lp12_0782	Putative glycosyltransferase
Lp6_766		Acetyltransferase	100		lp12_0783	Putative *N*-acetyltransferase
Lp6_767	HisF4	Imidazoleglycerol-phosphate synthase	100	HisF	lp12_0784	Putative imidazole glycerol phosphate synthase
Lp6_768	HisH2	Imidazole glycerol phosphate synthase	100	HisH	lp12_0785	Putative imidazole glycerol phosphate synthase
Lp6_769		LPS biosynthesis protein	100		lp12_0786	LPS biosynthesisprotein
Lp6_770		Aryl-alcohol dehydrogenase-like oxidoreductase	100		lp12_0787	Putative aldo/keto reductase
Lp6_771	DegT	Aminotransferase	100		lp12_0788	AHBA synthase
Lp6_772	CapD	Polysaccharide biosynthesis protein	100	CapD	lp12_0789	Putative polysaccharide biosynthesis protein
Lp6_773		HP	100		lp12_0790	HP
Lp6_774		Capsule polysaccharide biosynthesis protein	100		lp12_0791	HP
Lp6_775	NeuB	*N*-acetylneuraminic acid synthetase	100	NeuB	lp12_0792	Putative *N*-acetylneuramic acid synthetase
Lp6_776		HP	100		lp12_0793	Putative methyltransferase
Lp6_777		HP	100		lp12_0794	Putative aminopeptidase
Lp6_780		HP	100		lp12_0797	HP
Lp6_781		Dehydrogenase	100		lp12_0798	Putative dehydrogenase
Lp6_782		Dehydrogenase	100		lp12_0799	Oxidoreductase domain-containing protein
Lp6_825		HP	48	FtsY	lp12_2663	Cell division membrane protein
Lp6_828		UDP-*N*-acetyl-D-galactosamine dehydrogenase	100		lp12_0849	UDP-glucose/GDP-mannose dehydrogenase
Lp6_829		UDP-glucose 4-epimerase	100		lp12_0850	NAD dependent epimerase
Lp6_830		Starch synthase	100		lp12_0851	Putative Starch synthase
Lp6_831		HP	100		lp12_0852	TRP containing protein
Lp6_832		HP	100		lp12_0853	HP
Lp6_833		Glycosyl transferase family 2	100		lp12_0854	Glycosyl transferase
Lp6_976		HP	54		lp12_1008	Putative integrase
Lp6_977	YwfO	Phosphohydrolase	63		lp12_1057	Deoxyguanosine triphosphate triphosphohydrolase
Lp6_1186		HP	38		lp12_0204	Cytochrome D ubiquinol oxidase
Lp6_1249	XerD	Integrase/recombinase	62		lp12_2416	HP
Lp6_2002		integrase	100		lp12_2057	integrase
Lp6_2003		Protein of unknown function DUF1016	100		lp12_2058	HP
Lp6_2041		HP	100		lp12_2105	HP
Lp6_2164		HP	100		lp12_0810	HP

HP identifies hypothetical proteins.

We next defined a non-Sg1 core of 2946 proteins, by comparing Sg6 str. Thunder Bay and Sg12 str. 570-CO-H predicted proteins [72] (Table S3). Upon comparing with the Sg1 core, 435 proteins were defined as divergent from the non-Sg1 core. This included the O-antigen biosynthetic cluster, flagellar assembly, T4SS substrates and some metabolic enzymes. Interestingly, amongst the 2486 Sg1 core proteins, only 79 were defined as divergent compared to the non-Sg1 core (Table S4), and included genes from O-antigen biosynthetic cluster and the Lvh region of the type IV-A secretion system [73]. These differences might reflect the discrepancy in growth and replication in the environment or the human host.

### Differences in the O-antigen Structure of Sg1 Confer Resistance to the Alternative Complement Pathway

**Figure 6:**
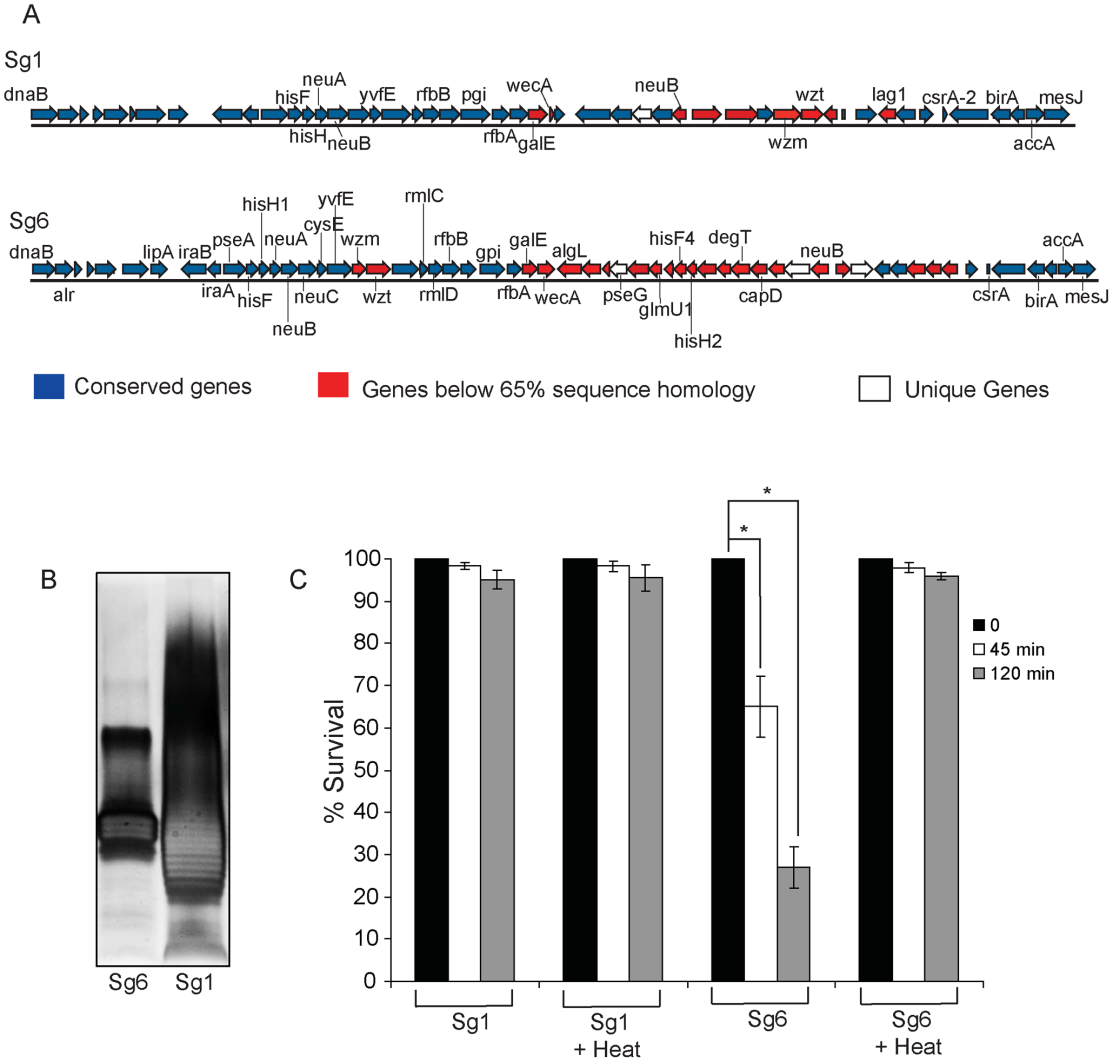
The O-antigen locus of *L. pneumophila*. (A) Layout of O-antigen region in Sg1 str. Philadelphia and Sg6 str. Thunder Bay in the area of highest dissimilarity, corresponding to the LPS gene cluster. (B) 16% SDS gel analysis of purified LPS from Sg1 str. Philadelphia and Sg6 str. Thunder Bay. (C) Comparative serum resistance of (Sg1) Sg1 str. Philadelphia and (Sg6) Sg6 str. Thunder Bay in Non-immune Human Serum. Presented data are an average of three independent experiments. *denotes statistical significance as determined by a two-tailed student’s t-test with a P-value <0.05.

Comparative genomics and optical mapping analysis of the Sg6 str. Thunder Bay genome revealed that the O-antigen biosynthetic proteins present major variations with respect to the Sg1str. Philadelphia (Figure 6A). Therefore, we predicted that there might be changes in the O-antigen generated by Sg1 str. Philadelphia and Sg6 str. Thunder Bay. To verify this hypothesis, purified lipopolysaccharide (LPS) from these strains were visualized through SDS-PAGE. Sg1 displayed a classic ladder pattern (Tsai and Frasch, 1982), which suggests that the O-antigen is highly decorated [74]. Conversely, the Sg6 O-antigen appears to be minimally decorated and lacked higher molecular weight species (Figure 6B). Our Sg6 str. Thunder Bay profile is in agreement with a previous study that analyzed LPS from Sg6 str. MP [75]. Previously, differences in O – antigen profiles have been used to define distinct serogroups [76], [77], and are hypothesized to be associated to differences in virulence of *L. pneumophila* serogroups in humans [42].

Variations in the outer-membrane O – antigen segment of the LPS can provide resistance to complement mediated serum killing in pathogenic bacteria [78], [79], [80]. The differences observed in the O – antigen structure of Sg1 str. Philadelphia and Sg6 str. Thunder Bay indicated that there might be differential sensitivity to serum complement. Indeed when the Sg6 was treated with Non-immune Human Serum (NHS), a 35% and a 73% decrease in colony forming units (CFUs) was seen by 45 and 120 min, respectively (Figure 6C). However, no effect was observed for Sg1. Sg6 and Sg1 viability was not affected by decomplemented (heat inactivated) NHS, consistent with a Sg6-specific killing by heat-sensitive complement. These results suggest that Sg6 O-antigen is unable to provide any appreciable resistance to serum complement, whereas Sg1 O-antigen may allow the bacteria to escape complement mediated killing by naive human serum.

**Figure 7:**
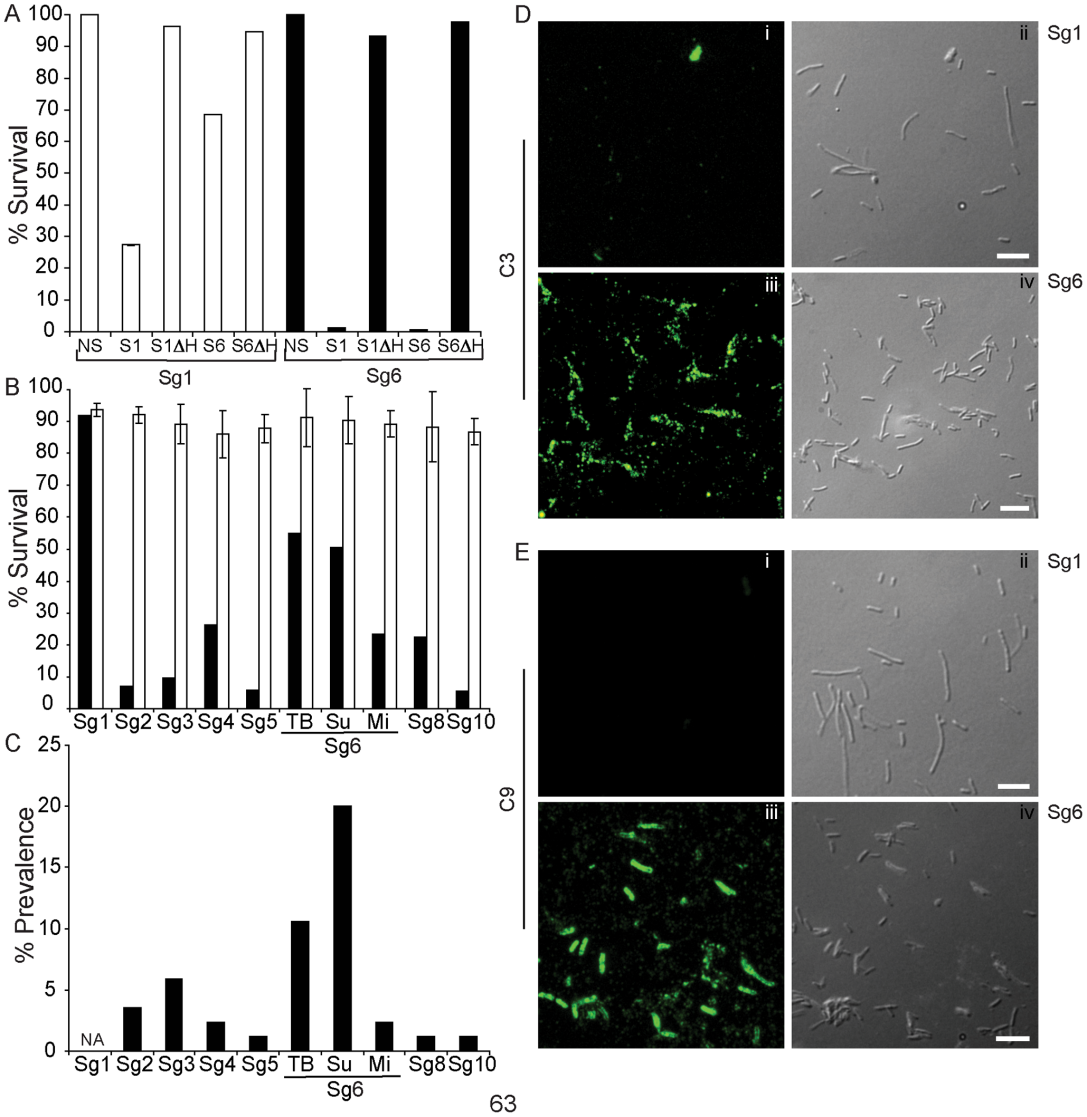
Serum Resistance of non-Sg1 *L. pneumophila*. (A) Percentage survival of Sg1 str. Philadelphia vs Sg6 str. Thunder Bay in the presence of no Serum (NS), serum with Sg1 antibodies (S1), S1 heated for 30 min at 56°C (S1ΔH), serum with Sg6 antibodies (S6) and S6 heated for 30 min at 56°C (S6ΔH). (B) Percentage survival of *L. pneumophila* serogroups after incubation in 90% non-immune human serum for 1 hour at 37°C. Both panels A and B are an average of three independent experiments. (C) Relative prevalence of non-SG1 in the clinical isolates database maintained at Public Health Ontario Laboratories. (D) Immunodetection of complement proteins C_3_ and (E) C_9_ at the surface of NHS treated Sg1 str. Philadelphia vs Sg6 str. Thunder Bay. Panels on the left are fluorescent captures of the differential interference contrast images on the right. The bacteria are seen as individual rods in these images. Images were acquired using Quorum Optigrid microscope with a 63× oil immersion objective (Leica DMI6000B stand with a Hamamatsu EM-1K, EMCCD camera). Image acquisition and post-acquisition processing was performed using Volocity 4.3 software (Improvision).

We next measured the sensitivity of Sg1 str. Philadelphia and Sg6 str. Thunder Bay to the classical complement pathway using sera obtained from patients diagnosed with either Sg1 or Sg6 infections (Figure 7A). Presence of either Sg1 or Sg6 specific antibodies was previously confirmed through IFA (data not shown). The Sg1 str. Philadelphia isolate showed a 72.5% decrease in CFU after a 60 min incubation in sera containing Sg1 antibodies, an effect that was not observed in decomplemented patient sera. In presence of Sg6 specific antibodies, the Sg1 str. Philadelphia CFUs were decreased by 31.5% at 60 min. The Sg6 str. Thunder Bay counts, when incubated with Sg1 antibodies were decreased by 98.8%, and similarly decreased by 99.3% in Sg6 patient sera. Taken together, these results suggest that while Sg1 str. Philadelphia is resistant to the alternate complement system, it is highly sensitive to the classical pathway of complement mediated killing. Furthermore, Sg1 are more sensitive to this pathway in the presence of Sg1 specific antibodies, while the Sg6 can be targeted by both Sg1 and Sg6 antibodies.

The above experiments suggested that the high prevalence of Sg1 in patient cases might be related to higher resistance to serum complement. We hypothesized that we might observe a similar correlation between clinical prevalence and resistance to the alternative complement pathway amongst the non-Sg1 strains in our repository. From this set, we selected the most prevalent sequence type for each serogroup, and measured their survival in NHS (Figure 7B). The highest CFU/ml counts were obtained for Sg6 strains Thunder Bay and Sudbury, which account for 20% and 10.6% of all non-Sg1 infections, respectively. Interestingly Sg6 str. Mississauga, which account for only 2.35% of all clinical cases, showed a marked reduction in survivability compared to Thunder Bay and Sudbury strains (Figure 7C). All other serogroups that shared similar prevalence as Sg6 str. Mississauga, exhibited high sensitivities to serum complement. These results strongly indicate that while all non-Sg1’s are sensitive to serum complement, the degree of susceptibility correlates strongly with clinical prevalence.

### Serum Complement Proteins are able to Bind to the Surface of Sg6 but not Sg1

Serogroup specific differences in complement resistance may be reflected in the efficiency to avoid the formation of membrane attack complex (MAC) [81], [82]. MAC forms transmembrane channels in the bacterial membrane, leading to depolarization and cell death [83]. We compared the binding of complement proteins C_3_ and C_9_ to the surfaces of Sg1 str. Philadelphia and Sg6 str. Thunder Bay by indirect fluorescent antibody (IFA). C_3_ protein is able to bind to the LPS of bacteria and provides the scaffold for the membrane attack complex [84], [85]. The binding of the C_9_ protein completes the membrane attack complex, which leads to bacterial death [85], [86], [87]. Our experiments indicate that while only residual amounts of C_3_ bind Sg1 (Figure 7D i), significant quantities were detected on the Sg6 surface (Figure 7D iii). This subsequently led to the detection of C_9_ on Sg6 (Figure 7E iii), but absent from Sg1 surfaces (Figure 7E i). This result suggests that the membrane attack complex can be assembled in Sg6 but not in Sg1, highlighting Sg6’s sensitivity to the alternative complement pathway.

### Sg1 Disseminates more Efficiently than Sg6 in the A/J Mice Infection Model

**Figure 8:**
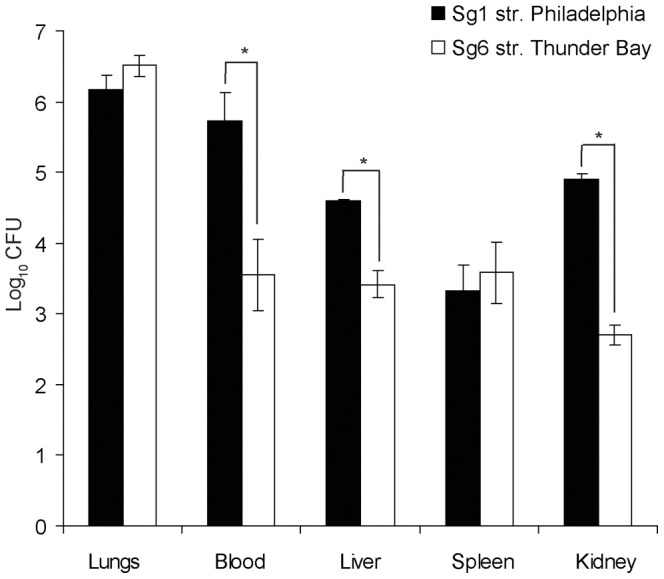
Sg1 and Sg6 infections in A/J mice. CFU counts of Sg1 and Sg6 in different organs and blood 48 h post-intratracheal inoculation. Both Sg1 and Sg6 groups consisted of 5 mice each.

The different serum resistance profiles of Sg1 and Sg6 suggested that these serogroups might differ in their ability to cause infection in the lungs and/or dissemination to other organs. In order to test this hypothesis, we infected A/J mice intratracheally with Sg1 str. Philadelphia or Sg6 str. Thunder Bay and determined the severity of the disease 48 hours post intra-tracheal inoculation [88], [89]. Forty eight hours post infection, mice infected (n = 5) with Sg1 showed symptoms of distress as indicated by ruffled fur, loss of appetite and lethargy. Conversely, mice infected with Sg6 (n = 5) displayed no deviation from normal behaviour and appearance. Furthermore, while no significant differences were seen in bacterial counts in the lungs, a 100-fold increase was noted for Sg1 in blood. Similarly, 100-fold and 10-fold increases were also seen for Sg1 in kidneys and liver, respectively (Figure 8). Surprisingly, no differences were observed in the spleens. This data suggests that while both Sg1 and Sg6 cause similar level of infections in the lung, they differ in their ability to induce bacteraemia and disseminate to other organs. Severe complications associated with Legionnaire’s disease are well documented and include septicemia and multi-organ failure [90], [91]. Therefore, our data are in agreement with the clinical manifestations of Legionnaire’s disease, and suggests that bloodstream dissemination of *L. pneumophila* is a key determining factor of the severity of legionellosis.

## Discussion

The human host provides a challenging environment for the survival of pathogens. Given that no human to human transmission has been reported for *L. pneumophila*, the clinical prevalence of *L. pneumophila* Sg1 over other serogroups poses a puzzling problem, since any Sg1 specific advantage in the human host must be a secondary consequence to an environmental benefit. Here, we thoroughly investigated this question by reporting a comprehensive study comparing the genomes, the clinical impact, the environmental fitness and the pathogenicity of a clinically prevalent Sg6 strain with a phylogenetically related Sg1 strain.

By combining MLVA and SBT analyses of a population based repository of non-Sg1 clinical isolates, we could identify a large cluster of prevalent molecular types presenting a low diversity. At the phylogenetic level, isolates from this cluster seem clonally stable as they have been collected for over 30 years. Based on this finding, a member of this cluster, Sg6 str. Thunder Bay, was selected for further analyses. Given the high plasticity of previously reported *Legionella* genomes, the optical maps of Sg6 str. Thunder Bay and Sg1 str. Philadelphia showed a high degree of similarity in genomic structure and restriction pattern, except for one large divergent region that includes several genes involved in O-antigen biosynthesis [49]. Furthermore, a comparison of Sg6 str. Thunder Bay and Sg12 str. 570-CO-H optical maps identified the O-antigen region to be conserved between these two sequenced non-Sg1 strains. Comparative genomics analysis was consistent with our optical mapping experiments, where a majority of genetic differences between Sg1 str. Philadelphia and Sg6 str. Thunder Bay were identified in the O-antigen biosynthetic cluster. The potential role of the LPS cluster in the predominance of Sg1 was initially hypothesised in a large multigenome microarray analysis of *L. pneumophila* strains [42]. More recently, the O-antigen regions of several serogroups were sequenced and compared using a PCR/Sanger sequencing approach. Using this approach, O-antigen biosynthetic genes *wzt*, *wzm* and *lag*1 were only identified in Sg1 and it was postulated that these genes might be absent in other serogroups [59]. Here, while at the protein level they only share low homology with the Sg1 and discrepancies with previous reports may be explained by the use of different comparative genomics cut-off values, next generation sequencing approaches allowed us to identify distant homologues *of wzm*, *wzt* and *galE* genes in Sg6. Together Wzm and Wzt form the inner membrane O-antigen transporter [92], [93] and GalE is a cytoplasmic epimerase that converts UDP-Galactose to UDP-Glucose upon which the remaining O-antigen sugars are assembled [94]. Lag1 was previously described as an O-acetyltransferase in some Sg1 strains [95], shared less than 50% sequence identity with a predicted AlgL an alginate acetyltransferase in all three Sg6 strains and Sg12 str. 570-CO-H. Although, mutations in *lag-1* have been associated with lack of the 8-*O*-acetyl groups in legionaminic acid leading to polymorphic changes in the LPS, these modifications were shown to have no effect on serum resistance or uptake in amoeba [96]. Interestingly, it has been proposed that the 8-*O*-acetylation of first three O-antigen sugars, which are located closest to the core sugars, might occur via a Lag1 independent pathway [95].

The chemical structure of Sg1 O-antigen is very unusual due to its atypical hydrophobic nature, owing to the presence of a homopolymer of legionaminic acid on its surface [97]. Previous analysis of the LPS from non-Sg1 suggested that *L. pneumophila* O-antigens are structurally conserved, since legionaminic acid was identified in all serogroups, although they lacked *O*-acetylation [98]. This finding was further substantiated when O-antigens from different *L. pneumophila* serogroups were visualized through SDS-PAGE, and polymorphic changes compared to Sg1 were reported [99]. Our analysis indicates that while Sg6 does generate an O-antigen, it might lack high molecular weight species.

The structural differences in the O-antigen leading to the increased clinical prevalence of Sg1 may be the result of a selective pressure from the natural bacterial reservoir. In other pathogens, O-antigen plays a role in biofilms architecture [100] and predation by environmental amoebae [101]. Furthermore, GalE, which showed serogroup specific differences in our study, has previously been shown in *Porphyromonas gingivalis* to play a significant role both in O-antigen biosynthesis and biofilm production [102]. In light of these studies, we hypothesized that the O-antigen differences between Sg1 and Sg6 might confer an advantage for Sg1 in surviving in the environment. Comparative analysis showed that Sg6 str. Thunder Bay generates increased levels of biofilm compared to Sg1 str. Philadelphia, suggesting that the Sg6 might possess an advantage when it comes to colonizing water systems. A second strategy employed by *L. pneumophila* to survive within environmental water systems is to replicate within the amoeba host. Although our analysis was limited to one reference amoebae species model [103], Sg1 str. Philadelphia and Sg6 str. Thunder Bay were equally effective in infecting *A. castellani*, while Sg6 str. Thunder Bay is able to replicate better within these amoebae. These findings, in combination with the conclusions drawn from our biofilm analyses, suggest that Sg1 may not possess environmental advantage upon other serogroups. Furthermore, the increased efficiency of Sg6 in replicating in amoebae should most likely permit its transmission in larger doses. This is in agreement with previous studies that have either detected similar levels of Sg1 and non-Sg1 in environmental surveys, or have reported the environmental prevalence of Sg6 over all serogroups [104], [105], [106], [107]. More specifically, studies report that Sg6 strains are more abundant in urban water sources in Ontario [53], and are also more prevalent than Sg1 strains in environmental niches, like the Great Lakes [108]. More so, in absence of an obvious demonstrated environmental advantage for Sg1 strains, it remains unknown why certain LPS structures have been selected, but we speculate it may protect Sg1 strains from being compromised by exposure to environmental stress factors.

In several pathogens, highly decorated O-antigens structures provide protection against serum complement [79], [109], [110]. Therefore, we hypothesized that different O-antigen profiles of Sg1 and Sg6 might confer different levels of resistance to serum complement. Our data indicates that Sg1 str. Philadelphia is highly resistant to the alternative complement system, while both Sg1 str. Philadelphia and Sg6 str. Thunder Bay are susceptible to the classical complement pathway. Interestingly, differences in serum resistance amongst different serogroups correlated to their clinical prevalence, since Sg6 str. Thunder Bay and Sudbury, the two most predominant non-Sg1 strains in Ontario exhibited the least sensitivity to the alternative complement system. *L. pneumophila*’s susceptibility to the classical complement pathway has previously been reported [111]. Unlike the classical complement pathway where complement proteins bind the surface of bacteria opsonised with IgG/IgM [112], in the alternative pathway the spontaneous hydrolysis of C_3_ into C_3a_ and C_3b_ fragments triggers the activation of the complement cascade [113]. C_3_ was previously shown to bind MOMP on *L. pneumophila* surfaces in small quantities, which may lead to the activation of the alternative complement system [114]. Perhaps, the highly decorated LPS of Sg1 interferes with this interaction, and therefore, would lead to a limited binding of C_3_, resulting in increased resistance to serum complement [115]. This is in agreement with previous evidence that suggests that certain strains of Sg1 might be resistant to alternative complement pathway as well [116]. A previous study also suggested that C_3_ cannot be detected on Sg1 surfaces using IFA in the absence of serum antibodies [117]. Similarly, we could not detect C_3_ on Sg1 str. Philadelphia surface, but it was present on the surface of Sg6 str. Thunder Bay. We also detected C_9_ on the surface Sg6 str. Thunder Bay indicating the activation of the complement cascade.

We next compared the virulence of Sg1 str. Philadelphia and Sg6 str. Thunder Bay using *in vitro* and *in vivo* experimental models. Both strains showed equal ability to infect and replicate within human lung epithelial cells. Unexpectedly, Sg6 str. Thunder Bay showed a high propensity to replicate inside macrophages compared to Sg1 str. Philadelphia. In intratracheally infected naïve mice, our findings indicate that both strains have comparable ability to colonize lungs, suggesting that complement mediated killing via the alternative pathway does not play a significant role in the immune response against *L. pneumophila* within lungs. Previously, the alternative complement cascade was shown to be limited within lungs [118]. From the lung alveoli, the bacteria may disseminate to other organ systems by crossing the lung endothelium as either free bacteria or inside macrophages [119]. Strikingly, Sg1 str. Philadelphia and Sg6 str. Thunder Bay differed in their potential to disseminate to other organs and to induce bacteraemia. In blood, where complement mediated lysis is most active, the Sg6 counts were 100-fold lower than Sg1. Significantly lower Sg6 counts were also seen in the liver and kidney. This is presumably due to the blood processing roles of these organs. The lower counts of Sg6 str. Thunder Bay in the blood suggests that after the first cycle of replication in circulating macrophages, the bacteria lyse the cells for a second round of infection, and in doing such exposes itself to the complement system. Surprisingly, no serogroup specific difference was seen in the spleen. Dissemination to kidneys and liver requires transit through blood, but both lymphatic and the circulatory system are directed to the spleen. Taking this into account, the data suggests that Sg6 and possibly *L. pneumophila* use the lymphatic system for dissemination beyond the lungs. All together our *in vitro* and *in vivo* results support that resistance to the alternative complement system may play a crucial role in the high clinical prevalence and severity of *L. pneumophila* Sg 1 infections.

The analysis of the role of O – antigen would be better examined by swapping the large O – antigen biosynthetic cluster between *L. pneumophila* Sg1 and Sg6. However, genetic tools to achieve this exchange have yet not been developed. Furthermore, the region in question has previously been shown to be an essential element in the genome of *L. pneumophila* [120], therefore limiting the likelihood of obtaining knockout mutants. Thus, the data presented in this comprehensive study combining long term molecular epidemiology, comparative genomics and pathogenicity analyses provide a unique perspective on the role of serogroups in *L. pneumophila* infections. The ability of Sg1 strains to tolerate the alternative complement cascade may allow it to disseminate to other organs via transit through blood, which is in agreement with the septicaemia and bacteraemia associated with the disease [90], [91]. Because there appears to be no immediate benefit of these differences within biofilms or a natural amoebal host, future investigations explaining why this specific trait is sequestered amongst the Sg1 strains will provide critical insight into the environmental factors and hosts that shape the evolution of this important pathogen’s virulence.

## Materials and Methods

### Ethics Statement

The collection of control human blood from healthy volunteers used in this study was approved by the Research Ethics Board of the Mount Sinai Hospital (Toronto, Canada). As approved by the Research Ethics Board of the Mount Sinai Hospital, a written informed consent to participate in this study was provided by all participants. The Keenan Research Centre of the Li Ka Shing Knowledge Institute, Animal Care and Use Committee reviewed and approved our study protocol for infecting and sampling mice with *Legionella pneumophila*. All work in our study was conducted adhering to the institution’s guidelines for animal husbandry, and followed the guidelines Canadian council on animal care for the care and use of Laboratory Animals.

### Strains used in this Work

Legionellosis is a notifiable disease in Ontario (population 13 million persons). Since 1978, the diagnosis of *Legionella* infections has been centralized at the Ontario Public Health Laboratory. This laboratory serves as the *Legionella* reference laboratory and performs all testing for outbreak investigations and most testing of clinical specimens. Therefore, isolates analyzed in this study are representative of the non-Sg1 strains isolated in Ontario in the past 3 decades. The proportion of culture-confirmed case-patients with *L. pneumophila* infection remained stable during the period of analysis, and 34% of the isolates were non-SG1 [39]. Species and serogroups were confirmed by direct immunofluorescent antibody assay and slide-agglutination [121], [122]. Isolates (n = 81) were stored at –80°C in trypticase soy broth supplemented with 5% horse blood. Eight isolates could not be resuscitated and were omitted from our analysis. With the exception of 2 Sg6 isolates collected at the same date in the same hospital, none of the isolates were epidemiologically related. *L. pneumophila* ST 39 (Sg2–3 culture confirmed cases), ST 68 (str. Sudbury (CG346); Sg6–9 culture confirmed cases), ST 187 (str. Thunder Bay (CG315); Sg6–17 culture confirmed cases), ST 378 (Sg4–2 culture confirmed cases), ST 407 (str. Mississauga (CG331); Sg6–2 culture confirmed cases), ST 465 (Sg3–5 culture confirmed cases), ST 466 (Sg8–1 culture confirmed case), ST 470 (Sg10–1 culture confirmed case), ST 471 (Sg5–1 culture confirmed case) were selected among the 73 typable isolates for further analyses. Reference clinical strain *L. pneumophila* subspecies pneumophila str. Philadelphia-1 (ST 36) was obtained from ATCC (#33152).

### Molecular Typing Schemes

The initial molecular typing of *L. pneumophila* strains was conducted using *Legionella* Sequence based Typing (SBT) scheme [43], [44], [123]. Molecular typing was also concurrently done through the Multiple-Locus VNTR Analysis Typing Scheme (MLVA), as formerly reported [45]. A combined MLST-MLVA dendrogram was also prepared according to a recently described method [46]. The serogroups of patient isolated strains was determined using a method previously described [39].

The Sg6 str. Thunder Bay optical map was generated according to the manufacturer’s protocol (OpGen). In short, the strain was grown for four days on buffered charcoal yeast extract (BCYE) agar at 37°C with 5% CO_2_. Genomic DNA was extracted using the OpGen DNA extraction kit from a single colony. DNA purification via this method generated concentrated, non-sheared DNA. The DNA was allowed to relax overnight and its quality was checked via a Q-Card. The DNA was next applied to a special cover slip using a wide-bore pipette, and as a result of capillary action the DNA was stretched in channels. The DNA was stained with a fluorescent dye and in the presence of an antifade solution, and was visualized through a laser microscope. The DNA concentration was adjusted to achieve 20–30 DNA fragments/frame, with a minimum size of 150 kB. The diluted DNA was applied to the M-card, and was treated with *Nhe*I for 30 min. The digested pieces of DNA were visualized via the Optical mapping array and fragment length was assigned by the onboard software (MapManager). These fragments were aligned to generate a circularized map with a minimum coverage of 65 X. Optical maps for reference strains were constructed *in silico* by providing genome sequences from public databases. The optical maps were used to construct an evolutionary tree using Mapsolver (OpGen) based on the UPGMA algorithm.

### *In vitro* Biofilm Assays

Biofilm assays were conducted according to a previously published protocol [124]. Briefly, single clones grown for 4 days on BCYE agar at 37°C and 5% CO_2_ were used to inoculate broth cultures, which were allowed to reach an OD of 2.0 and diluted in fresh medium to give a final OD at 600 nm of 0.2. In a 96 well plate, 200 µl of this suspension was dispensed, and the plates were incubated at 37°C and 5% CO_2_ for 2 and 4 days. Biofilms were stained with 40 µl 0.25% crystal violet in each well for 15 min and washed 3 times in deionized water, and the crystal violet stain was solubilized in 95% ethanol. Absorbance was read at 600 nm. Three independent experiments were performed using eight replicates.

### *Acanthamoeba castellani* and U937 Derived Macrophage Inoculations

*A. castellan*i was obtained from ATCC (50739) and was cultured in peptone yeast glucose (PYG) broth (2% Peptone, 0.1% Yeast Extract, 0.1 M Glucose, 4 mM MgSO_4_, 0.4 M CaCl_2_, 0.1% HOC(COONa)(CH_2_COONa)_2_ · 2H_2_O, 0.05 mM Fe(NH_4_)_2_(SO_4_)_2_-6H2O, 2.5 mM NaH_2_PO_3_, 2.5 mM K_2_HPO_3_, pH 6.5) as previously described [125]. U937 monocytes were obtained from ATCC (CRL-1593.2) and cultured in Roswell Park Memorial Institute (RPMI) media, supplemented with Glutamine and 10% heat inactivated fetal bovine serum. All cells were passaged 3 times before inoculation assays were carried out. U937 cells were differentiated into adherent macrophages by treatment with 1.0×10^−3^ mg/ml 12-O-Tetradecanoylphorbol-13-Acetate for 48 hours. *L. pneumophila* Sg1 and Sg6 patches were grown on BCYE agar for 4 days at 37°C with 5% CO_2_ and suspended in PBS. In a 24 well plate, 10^5^ amoebae/macrophages were incubated with 10^6^ bacteria for a multiplicity of infection (MOI) of 10. The incubation was carried out for 2 hours at 37°C with 5% CO_2_, after which free bacterial cells were removed by washing the wells 3 times with AC buffer (4 mM MgSO_4_, 0.4 M CaCl_2_, 0.1% HOC(COONa)(CH_2_COONa)_2_ · 2H_2_O, 0.05 mM Fe(NH_4_)_2_(SO_4_)_2_-6H_2_O, 2.5 mM NaH_2_PO_3_, 2.5 mM K_2_HPO_3_, pH 6.5) or PBS. Next the infected cells were treated with gentamicin (100 µg/ml) for 1 hour to kill extracellular bacteria and synchronize the infections. The amoebae/macrophages were washed 3 times with AC buffer/PBS and incubated for 0, 24 and 48 hours in AC buffer/PBS at 37°C with 5% CO_2_. At the end of the incubation period the cells were lysed by incubating them for 5 min in ice-cold filter sterilized tap water, followed by successive passes through a 27.5 gauge needle. Serial dilutions of lysate and supernatant mixture were plated on BCYE agar and incubated for 4 days at 37°C with 5% CO_2_ for CFU enumeration.

### NCI-H292 Lung Epithelial Cell Infections

Binding and invasion assays of NCI-H292 (ATCC, CRL-1848) cells from human lung mucoepidermoid carcinoma with *L. pneumophila* str. Philadelphia and Thunder Bay were conducted as previously described [124], but with the following modifications: Bacteria were resuspended in PBS from 4 day old plates that had been incubated at 37°C with 5% CO_2_, and lung epithelial cells were inoculated at an MOI of 10 and were lysed by incubation in filter sterilized tap water for 5 minutes and passed through a 27.5 gauge needle.

### LPS Purification and O-antigen Analysis

LPS were purified from *L. pneumophila* str. Philadelphia and Thunder Bay as previously described [99]. In brief, bacterial suspensions (OD_600_ 2.0) were centrifuged at 10,000×g for 5 min. The pellets were washed twice in 5 ml PBS (pH 7.2) containing 0.15 mM CaCl_2_ and 0.5 mM MgCl_2_. Pellets were then resuspended in 10 ml PBS (pH 7.2) and sonicated at 4 W three times at an output setting of 0.5 (Misonix S3000) for 10 min on ice. In order to eliminate contaminating protein and nucleic acids, samples were treated with proteinase K (100 µg/mL) at 65°C for 2 hours, followed by DNaseI (20 µg/mL) and RNase (40 µg/mL) treatments at 37°C overnight. The extraction of LPS was initiated by adding an equal volume of hot (65–70°C) 90% phenol with vigorous shaking at 65–70°C for 2 hours. Suspensions were cooled on ice, transferred to 1.5 mL polypropylene tubes and centrifuged at 8500×g for 15 min. Supernatants were transferred into 15 mL conical centrifuge tubes and phenol phases were re-extracted using 300 µL dH_2_O. Sodium acetate at 0.5 M final concentration followed by 10 volumes of 95% ethanol were added to the extracts and samples were stored at −20°C overnight in order to precipitate LPS. Tubes were centrifuged at 2000×g, 4°C for 10 min and the pellets were resuspended in 1 ml distilled water. Extensive dialysis against distilled water at 4°C was carried out to remove any residual phenol. Purified LPS was solubilized in Laemmli buffer and boiled for 5 min to achieve a final concentration of 1 mg/ml. 20 µL/well from each sample was separated on 15% SDS gel with a 4% stacking gel under reducing condition at 100 mA for 2 hrs, using a mini-PROTEAN electrophoresis instrument (Bio-Rad Laboratories). Silver staining of the gels was performed according to the manufacturer’s protocol (Bio-Rad Laboratories).

### DNA Extraction, Sequencing and Genome Assembly

The genome of *L. pneumophila* str. Thunder Bay was sequenced using a combination of Sanger, Roche-454 and Illumina sequencing platforms. All general aspects of Roche-454 library construction and sequencing were conducted according to the manufacturer’s directions. Preparation of Illumina libraries and sequencing were conducted as previously described [126]. Sequencing using the Roche-454 platform generated individual reads at a minimum 19× coverage, while Illumina paired-end reads were obtained at 99× coverage. Initial genome assembly of 454 reads via the Newbler software package (Roche) generated 36 large contigs. The order of the contigs was determined via optical mapping and gaps between these contigs were closed via primer walking to generate a single molecule. A total of 440 Sanger finishing reads were produced to close gaps, while Illumina reads were used to resolve repetitive regions, and raise the quality of the finished sequence. Sequencing errors were corrected by mapping Illumina reads to the assembled *L. pneumophila* str. Thunder Bay genome using CLC Genomics Workbench (Ver 5.1.1). single 454 contig. The closed genome was annotated through the GenDB platform. All gene annotations calls were checked manually and corrected based on greater than 95% homology with other *L. pneumophila* homologs, Pfam domains, TIGRFAM domains and SignalP predictions. Raw reads of Sg6 str. Sudbury (ST 68) and Mississauga (ST 407) were *de novo* assembled using CLC Genomics Workbench (Ver 5.1.1), and were transitively annotated against *L. pneumophila* str. Thunder Bay using the GenDB platform. The *L. pneumophila* str. Sudbury and *L. pneumophila* str. Mississauga genomes were compared with *L. pneumophila* str. Thunder Bay using GenDB (ver 5.1.1) for Single Nucleotide Polymorphism (SNP) analysis and gene content.

### Genome Annotation and Comparative Genomics

The single chromosome of *L. pneumophila* str. Thunder Bay was annotated using a modified version of the GenDB platform as previously described [127], [128]. In short, ORF prediction was done in GenDB using REGANOR [129], which uses CRITICA [130] and Glimmer [131] in addition to RBSFinder [132] to make ribosomal binding site and gene predictions. Ribosomal predictions are done using tRNA-Scan-SE [133] and RNAmmer [134]. All intergenic regions were elongated with a 25 base pair overlap on each end and run through a pipeline for identification of potential frameshifts and short genes overlooked by GenDB regional prediction pipeline. Regions found with significant BLASTX [135] hits to EMBL database [136] were identified, added as new CDS regions in GenDB. All coding regions were ran through the functional annotation pipeline by running a BLAST of each region against the following databases: PFAM [137], TIGRFAM [138], KEGG [139], NCBI’s non-redundant protein database, TMHMM [140], and SignalP [141].

Comparative proteomics was conducted by first comparing the *L. pneumophila* str. Thunder Bay translated genes against Sg1 core, which has previously been described [58]. Predicted proteins of *L. pneumophila* str. Thunder Bay that exhibited greater than 65% sequence identity were defined as the conserved group. These were then compared against the entire public database of known *L. pneumophila* proteins, in order to establish a list of unique proteins of *L. pneumophila* str. Thunder Bay. Furthermore, a list of unique proteins of the Sg1 core was compiled, by including proteins which showed less than 65% homology to *L. pneumophila* str. Thunder Bay predicted proteins. The pangenome of *L. pneumophila* was created using GView ver 1.6 [142].

### Serum Resistance Assays

Human blood was collected from 4 healthy volunteers and allowed to coagulate at room temperature for 30 min. The plasma was collected by centrifuging the tubes for 10 min at 1000×g and aspirating the supernatant. The plasma was analyzed via immunofluorescence assays (IFA) to confirm absence of *L. pneumophila* antibodies against Sg1 and Sg6 [143], and then was flash frozen in liquid nitrogen for storage at −80°C. Similarly, IFA was used to confirm the presence of Sg1 and Sg6 specific antibodies in patients diagnosed with Legionnaire’s disease.

*L. pneumophila* str. Philadelphia and Thunder Bay were grown for 4 days on BCYE agar at 37°C with 5% CO_2_. The bacteria were suspended in PBS to a final concentration of 10^6^ CFU/ml. The suspensions were diluted 10-fold in 90% non- immune human serum (NHS) and incubated at 37°C for 45 min and 90 min. In the experiments comparing serum resistance of several serogroups, a single incubation time of 60 min was selected. Similarly, experiments involving immune human serum (IHS) were carried out for 60 min. As controls, bacteria were also incubated with heat inactivated serum for the corresponding periods. Next bacteria were pelleted and washed 3 X with PBS, and CFU were determined by plating several serial dilutions on BCYE agar and incubated as before. These CFU values were compared to the inoculums and the data is reported as % survival.

### Immuno-fluorescence Asssays (IFA)

*L. pneumophila* str. Philadelphia and Thunder Bay were grown for 4 days on BCYE agar at 37°C with 5% CO_2_, were resuspended in 1× PBS and adjusted to OD 2.0. Bacteria were incubated in NHS for 25 min at 37°C and washed 2× with PBS. Bacterial suspensions were smeared onto glass slides and allowed to air dry. The smears were then heat fixed, followed by fixation in 4% paraformaldehyde for 15 min. Cells were blocked in 5% fetal calf serum in PBS for 1 hour at room temperature, followed by a 1 hour incubation with C_3_, C_5_ and C_9_ primary antibodies diluted (1∶10) in the blocking solution (Hycult Biotech). Images were acquired using Quorum tech Canada Optigrid structure illumination system in a Leica DMI6000B stand with a with a Hamamatsu EM-1K, EMCCD camera.

### A/J Mice Model

Six to eight week old female A/J mice (Jackson Laboratories) were infected with 50 µl of 10^6^ bacterial suspensions of either Sg1 str. Philadelphia or Sg6 str. Thunder Bay as previously described (Brieland et al., 1994). The bacterial suspensions were prepared in PBS from bacteria grown on BCYE agar plates for 4 days at 37°C with 5% CO_2_. The mice were infected via intra-tracheal inoculations. Forty eight hours post infection, the mice were sacrificed and organs were harvested. Homogenized organs and blood were plated on BCYE agar plates and grown for 4 days at 37°C with 5% CO_2_. The counts are reported as log_10_ CFU. The experiment was conducted in accordance with the approved protocol at the research vivarium of St. Michael’s hospital (Toronto, Ontario).

## Supporting Information

Figure S1 **SBT typing of a population based clinical repository of** ***L. pneumophila*** **.** Phylogenetic clusters were constructed based on UPGMA analysis of SBT distribution. The large cluster and the subclusters 1 and 2 are identified in the phylogenetic tree.
(PDF)

Figure S2 **MLVA typing of a population based clinical repository of** ***L. pneumophila*** **.** Phylogenetic clusters were constructed based on UPGMA analysis of MLVA based distribution. The large cluster and the subclusters 1 and 2 are identified in the phylogenetic tree.
(PDF)

Table S1 **General features of the sequenced Sg6 strains.**
(PDF)

Table S2 **Comparative proteomics analysis between Sg1 core genes and Sg6 str. Thunder Bay.** Proteins that share less than 65% sequence homology and 75% coverage were defined as divergent. HP identifies hypothetical proteins.
(DOC)

Table S3 **Genes that define the non-Sg1 core based on comparisons of Sg6 str. Thunder Bay and Sg12 str. 570-CO-H**.
(XLS)

Table S4 **Comparison of non-Sg1 core with the Sg1 core.**
(XLS)

## References

[1] Fraser DW, Tsai TR, Orenstein W, Parkin WE, Beecham HJ, et al (1977) Legionnaires' disease: description of an epidemic of pneumonia. N Engl J Med 297: 1189–1197. 335244 10.1056/NEJM197712012972201

[2] Newton HJ, Ang DK, van Driel IR, Hartland EL (2010) Molecular pathogenesis of infections caused by *Legionella pneumophila* . Clin Microbiol Rev 23: 274–298. 20375353 10.1128/CMR.00052-09 PMC2863363

[3] Winn WC Jr (1988) Legionnaires disease: historical perspective. Clin Microbiol Rev 1: 60–81. 3060246 10.1128/cmr.1.1.60 PMC358030

[4] Sanford JP (1979) Legionnaires' disease: one person's perspective. Ann Intern Med 90: 699–703. 373555 10.7326/0003-4819-90-4-699

[5] McDade JE, Shepard CC, Fraser DW, Tsai TR, Redus MA, et al (1977) Legionnaires' disease: isolation of a bacterium and demonstration of its role in other respiratory disease. N Engl J Med 297: 1197–1203. 335245 10.1056/NEJM197712012972202

[6] Yu VL, Plouffe JF, Pastoris MC, Stout JE, Schousboe M, et al (2002) Distribution of *Legionella* species and serogroups isolated by culture in patients with sporadic community-acquired legionellosis: an international collaborative survey. J Infect Dis 186: 127–128. 12089674 10.1086/341087

[7] Carratala J, Garcia-Vidal C (2010) An update on *Legionella* . Curr Opin Infect Dis 23: 152–157. 20051846 10.1097/QCO.0b013e328336835b

[8] Skaza AT, Beskovnik L, Storman A, Kese D, Ursic S (2012) Epidemiological investigation of a legionellosis outbreak in a Slovenian nursing home, August 2010. Scand J Infect Dis 44: 263–269. 22339541 10.3109/00365548.2011.635313

[9] Haupt TE, Heffernan RT, Kazmierczak JJ, Nehls-Lowe H, Rheineck B, et al (2012) An outbreak of legionnaires disease associated with a decorative water wall fountain in a hospital. Infect Control Hosp Epidemiol 33: 185–191. 22227989 10.1086/663711

[10] Silk BJ, Moore MR, Bergtholdt M, Gorwitz RJ, Kozak NA, et al.. (2012) Eight years of Legionnaires' disease transmission in travellers to a condominium complex in Las Vegas, Nevada. Epidemiol Infect: 1–10. 10.1017/S0950268811002779 22214820

[11] Legionellosis – United States, 2000–2009. MMWR Morb Mortal Wkly Rep 60: 1083–1086. 21849965

[12] Hicks LA, Garrison LE, Nelson GE, Hampton LM (2012) Legionellosis–United States, 2000–2009. Am J Transplant 12: 250–253. 22244124 10.1111/j.1600-6143.2011.03938.x

[13] Fields BS, Benson RF, Besser RE (2002) *Legionella* and Legionnaires' disease: 25 years of investigation. Clin Microbiol Rev 15: 506–526. 12097254 10.1128/CMR.15.3.506-526.2002 PMC118082

[14] Straus WL, Plouffe JF, File TM Jr, Lipman HB, Hackman BH, et al (1996) Risk factors for domestic acquisition of legionnaires disease. Ohio legionnaires Disease Group. Arch Intern Med 156: 1685–1692. 8694667

[15] Stout JE, Yu VL, Yee YC, Vaccarello S, Diven W, et al (1992) *Legionella pneumophila* in residential water supplies: environmental surveillance with clinical assessment for Legionnaires' disease. Epidemiol Infect 109: 49–57. 1499672 PMC2272241

[16] Stout JE, Yu VL, Muraca P, Joly J, Troup N, et al (1992) Potable water as a cause of sporadic cases of community-acquired legionnaires' disease. N Engl J Med 326: 151–155. 1727545 10.1056/NEJM199201163260302

[17] Leoni E, Legnani PP, Bucci Sabattini MA, Righi F (2001) Prevalence of *Legionella spp*. in swimming pool environment. Water Res 35: 3749–3753. 11561639 10.1016/s0043-1354(01)00075-6

[18] Tobin JO, Bartlett CL, Waitkins SA, Barrow GI, Macrae AD, et al (1981) Legionnaires' disease: further evidence to implicate water storage and distribution systems as sources. Br Med J (Clin Res Ed) 282: 573. 10.1136/bmj.282.6263.573 PMC1504294 6780130

[19] Costa J, Tiago I, da Costa MS, Verissimo A (2005) Presence and persistence of *Legionella* spp. in groundwater. Appl Environ Microbiol 71: 663–671. 15691915 10.1128/AEM.71.2.663-671.2005 PMC546754

[20] Rogers J, Keevil CW (1992) Immunogold and fluorescein immunolabelling of *Legionella pneumophila* within an aquatic biofilm visualized by using episcopic differential interference contrast microscopy. Appl Environ Microbiol 58: 2326–2330. 1637168 10.1128/aem.58.7.2326-2330.1992 PMC195776

[21] Mampel J, Spirig T, Weber SS, Haagensen JA, Molin S, et al (2006) Planktonic replication is essential for biofilm formation by *Legionella pneumophila* in a complex medium under static and dynamic flow conditions. Appl Environ Microbiol 72: 2885–2895. 16597995 10.1128/AEM.72.4.2885-2895.2006 PMC1448985

[22] Berk SG, Faulkner G, Garduno E, Joy MC, Ortiz-Jimenez MA, et al (2008) Packaging of live *Legionella pneumophila* into pellets expelled by *Tetrahymena spp*. does not require bacterial replication and depends on a Dot/Icm-mediated survival mechanism. Appl Environ Microbiol 74: 2187–2199. 18245233 10.1128/AEM.01214-07 PMC2292602

[23] Molmeret M, Horn M, Wagner M, Santic M, Abu Kwaik Y (2005) Amoebae as training grounds for intracellular bacterial pathogens. Appl Environ Microbiol 71: 20–28. 15640165 10.1128/AEM.71.1.20-28.2005 PMC544274

[24] Rowbotham TJ (1980) Preliminary report on the pathogenicity of *Legionella pneumophila* for freshwater and soil amoebae. J Clin Pathol 33: 1179–1183. 7451664 10.1136/jcp.33.12.1179 PMC1146371

[25] Abu Kwaik Y, Gao LY, Stone BJ, Venkataraman C, Harb OS (1998) Invasion of protozoa by Legionella pneumophila and its role in bacterial ecology and pathogenesis. Appl Environ Microbiol 64: 3127–3133. 9726849 10.1128/aem.64.9.3127-3133.1998 PMC106699

[26] Berk SG, Ting RS, Turner GW, Ashburn RJ (1998) Production of respirable vesicles containing live *Legionella pneumophila* cells by two *Acanthamoeba spp* . Appl Environ Microbiol 64: 279–286. 9435080 10.1128/aem.64.1.279-286.1998 PMC124706

[27] Brieland JK, Fantone JC, Remick DG, LeGendre M, McClain M, et al (1997) The role of *Legionella pneumophila*-infected *Hartmannella vermiformis* as an infectious particle in a murine model of Legionnaire's disease. Infect Immun 65: 5330–5333. 9393834 10.1128/iai.65.12.5330-5333.1997 PMC175767

[28] Shi C, Pamer EG (2011) Monocyte recruitment during infection and inflammation. Nat Rev Immunol 11: 762–774. 21984070 10.1038/nri3070 PMC3947780

[29] Horwitz MA, Silverstein SC (1980) Legionnaires' disease bacterium (*Legionella pneumophila*) multiples intracellularly in human monocytes. J Clin Invest 66: 441–450. 7190579 10.1172/JCI109874 PMC371671

[30] Horwitz MA (1983) The Legionnaires' disease bacterium (*Legionella pneumophila*) inhibits phagosome-lysosome fusion in human monocytes. J Exp Med 158: 2108–2126. 6644240 10.1084/jem.158.6.2108 PMC2187157

[31] Kagan JC, Roy CR (2002) *Legionella* phagosomes intercept vesicular traffic from endoplasmic reticulum exit sites. Nat Cell Biol 4: 945–954. 12447391 10.1038/ncb883

[32] Vogel JP, Andrews HL, Wong SK, Isberg RR (1998) Conjugative transfer by the virulence system of *Legionella pneumophila* . Science 279: 873–876. 9452389 10.1126/science.279.5352.873

[33] Segal G, Purcell M, Shuman HA (1998) Host cell killing and bacterial conjugation require overlapping sets of genes within a 22-kb region of the *Legionella pneumophila* genome. Proc Natl Acad Sci U S A 95: 1669–1674. 9465074 10.1073/pnas.95.4.1669 PMC19142

[34] Helbig JH, Luck PC, Witzleb W (1994) Serogroup-specific and serogroup-cross-reactive epitopes of *Legionella pneumophila* . Zentralbl Bakteriol 281: 16–23. 7528581 10.1016/s0934-8840(11)80632-8

[35] Luck PC, Helbig JH, Ehret W, Ott M (1995) Isolation of a *Legionella pneumophila* strain serologically distinguishable from all known serogroups. Zentralbl Bakteriol 282: 35–39. 7734827 10.1016/s0934-8840(11)80794-2

[36] Olsen CW, Elverdal P, Jorgensen CS, Uldum SA (2009) Comparison of the sensitivity of the *Legionella* urinary antigen EIA kits from Binax and Biotest with urine from patients with infections caused by less common serogroups and subgroups of *Legionella* . Eur J Clin Microbiol Infect Dis 28: 817–820. 19198903 10.1007/s10096-008-0697-x

[37] Helbig JH, Uldum SA, Luck PC, Harrison TG (2001) Detection of *Legionella pneumophila* antigen in urine samples by the BinaxNOW immunochromatographic assay and comparison with both Binax *Legionella* Urinary Enzyme Immunoassay (EIA) and Biotest *Legionella* Urin Antigen EIA. J Med Microbiol 50: 509–516. 11393288 10.1099/0022-1317-50-6-509

[38] Svarrer CW, Luck C, Elverdal PL, Uldum SA (2012) Immunochromatic kits Xpect *Legionella* and BinaxNOW *Legionella* for detection of *Legionella pneumophila* urinary antigen have low sensitivities for the diagnosis of Legionnaires' disease. J Med Microbiol 61: 213–217. 21921112 10.1099/jmm.0.035014-0

[39] Ng V, Tang P, Jamieson F, Guyard C, Low DE, et al (2009) Laboratory-based evaluation of legionellosis epidemiology in Ontario, Canada, 1978 to 2006. BMC Infect Dis 9: 68. 19460152 10.1186/1471-2334-9-68 PMC2695468

[40] Helbig JH, Bernander S, Castellani Pastoris M, Etienne J, Gaia V, et al (2002) Pan-European study on culture-proven Legionnaires' disease: distribution of *Legionella pneumophila* serogroups and monoclonal subgroups. Eur J Clin Microbiol Infect Dis 21: 710–716. 12415469 10.1007/s10096-002-0820-3

[41] Doleans A, Aurell H, Reyrolle M, Lina G, Freney J, et al (2004) Clinical and environmental distributions of *Legionella* strains in France are different. J Clin Microbiol 42: 458–460. 14715805 10.1128/JCM.42.1.458-460.2004 PMC321724

[42] Cazalet C, Jarraud S, Ghavi-Helm Y, Kunst F, Glaser P, et al (2008) Multigenome analysis identifies a worldwide distributed epidemic *Legionella pneumophila* clone that emerged within a highly diverse species. Genome Res 18: 431–441. 18256241 10.1101/gr.7229808 PMC2259107

[43] Gaia V, Fry NK, Afshar B, Luck PC, Meugnier H, et al (2005) Consensus sequence-based scheme for epidemiological typing of clinical and environmental isolates of *Legionella pneumophila* . J Clin Microbiol 43: 2047–2052. 15872220 10.1128/JCM.43.5.2047-2052.2005 PMC1153775

[44] Ratzow S, Gaia V, Helbig JH, Fry NK, Luck PC (2007) Addition of *neu*A, the gene encoding N-acylneuraminate cytidylyl transferase, increases the discriminatory ability of the consensus sequence-based scheme for typing *Legionella pneumophila* serogroup 1 strains. J Clin Microbiol 45: 1965–1968. 17409215 10.1128/JCM.00261-07 PMC1933043

[45] Visca P, D'Arezzo S, Ramisse F, Gelfand Y, Benson G, et al (2011) Investigation of the population structure of *Legionella pneumophila* by analysis of tandem repeat copy number and internal sequence variation. Microbiology 157: 2582–2594. 21622529 10.1099/mic.0.047258-0

[46] Sobral D, Le Cann P, Gerard A, Jarraud S, Lebeau B, et al (2011) High-throughput typing method to identify a non-outbreak-involved *Legionella pneumophila* strain colonizing the entire water supply system in the town of Rennes, France. Appl Environ Microbiol 77: 6899–6907. 21821761 10.1128/AEM.05556-11 PMC3187078

[47] Giongo A, Tyler HL, Zipperer UN, Triplett EW (2010) Two genome sequences of the same bacterial strain, *Gluconacetobacter diazotrophicus* PAl 5, suggest a new standard in genome sequence submission. Stand Genomic Sci 2: 309–317. 21304715 10.4056/sigs.972221 PMC3035290

[48] Latreille P, Norton S, Goldman BS, Henkhaus J, Miller N, et al (2007) Optical mapping as a routine tool for bacterial genome sequence finishing. BMC Genomics 8: 321. 17868451 10.1186/1471-2164-8-321 PMC2045679

[49] Ramirez MS, Adams MD, Bonomo RA, Centron D, Tolmasky ME (2011) Genomic analysis of *Acinetobacter baumannii* A118 by comparison of optical maps: identification of structures related to its susceptibility phenotype. Antimicrob Agents Chemother 55: 1520–1526. 21282446 10.1128/AAC.01595-10 PMC3067174

[50] Shukla SK, Kislow J, Briska A, Henkhaus J, Dykes C (2009) Optical mapping reveals a large genetic inversion between two methicillin-resistant *Staphylococcus aureus* strains. J Bacteriol 191: 5717–5723. 19542272 10.1128/JB.00325-09 PMC2737957

[51] Lau HY, Ashbolt NJ (2009) The role of biofilms and protozoa in *Legionella* pathogenesis: implications for drinking water. J Appl Microbiol 107: 368–378. 19302312 10.1111/j.1365-2672.2009.04208.x

[52] Wingender J, Flemming HC (2011) Biofilms in drinking water and their role as reservoir for pathogens. Int J Hyg Environ Health 214: 417–423. 21697011 10.1016/j.ijheh.2011.05.009

[53] Dutka BJ, Walsh K, Ewan P, El-Shaarawi A, Tobin RS (1984) Incidence of *Legionella* organisms in selected Ontario (Canada) cities. Sci Total Environ 39: 237–249. 6523127 10.1016/0048-9697(84)90081-0

[54] Barker J, Brown MR (1994) Trojan horses of the microbial world: protozoa and the survival of bacterial pathogens in the environment. Microbiology 140 (Pt 6): 1253–1259. 10.1099/00221287-140-6-1253 8081490

[55] Philippe C, Blech MF, Hartemann P (2006) Intra-amoebal development of *Legionella pneumophila* and the potential role of amoebae in the transmission of Legionnaires' disease. Med Mal Infect 36: 196–200. 16459041 10.1016/j.medmal.2005.10.010

[56] Jules M, Buchrieser C (2007) *Legionella pneumophila* adaptation to intracellular life and the host response: clues from genomics and transcriptomics. FEBS Lett 581: 2829–2838. 17531986 10.1016/j.febslet.2007.05.026

[57] Arakawa K, Tomita M (2007) The GC skew index: a measure of genomic compositional asymmetry and the degree of replicational selection. Evol Bioinform Online 3: 159–168. 19461976 PMC2684130

[58] D'Auria G, Jimenez-Hernandez N, Peris-Bondia F, Moya A, Latorre A (2010) *Legionella pneumophila* pangenome reveals strain-specific virulence factors. BMC Genomics 11: 181. 20236513 10.1186/1471-2164-11-181 PMC2859405

[59] Merault N, Rusniok C, Jarraud S, Gomez-Valero L, Cazalet C, et al (2011) Specific real-time PCR for simultaneous detection and identification of *Legionella pneumophila* serogroup 1 in water and clinical samples. Appl Environ Microbiol 77: 1708–1717. 21193672 10.1128/AEM.02261-10 PMC3067292

[60] Gillespie JJ, Ammerman NC, Dreher-Lesnick SM, Rahman MS, Worley MJ, et al (2009) An anomalous type IV secretion system in *Rickettsia* is evolutionarily conserved. PLoS One 4: e4833. 19279686 10.1371/journal.pone.0004833 PMC2653234

[61] Sexton JA, Vogel JP (2002) Type IVB secretion by intracellular pathogens. Traffic 3: 178–185. 11886588 10.1034/j.1600-0854.2002.030303.x

[62] Christie PJ, Vogel JP (2000) Bacterial type IV secretion: conjugation systems adapted to deliver effector molecules to host cells. Trends Microbiol 8: 354–360. 10920394 10.1016/s0966-842x(00)01792-3 PMC4847720

[63] Segal G, Russo JJ, Shuman HA (1999) Relationships between a new type IV secretion system and the icm/dot virulence system of *Legionella pneumophila* . Mol Microbiol 34: 799–809. 10564519 10.1046/j.1365-2958.1999.01642.x

[64] Land AD, Winkler ME (2011) The requirement for pneumococcal MreC and MreD is relieved by inactivation of the gene encoding PBP1a. J Bacteriol 193: 4166–4179. 21685290 10.1128/JB.05245-11 PMC3147673

[65] Wachi M, Doi M, Okada Y, Matsuhashi M (1989) New *mre* genes *mre*C and *mre*D, responsible for formation of the rod shape of *Escherichia coli* cells. J Bacteriol 171: 6511–6516. 2687239 10.1128/jb.171.12.6511-6516.1989 PMC210540

[66] Luneberg E, Zetzmann N, Alber D, Knirel YA, Kooistra O, et al (2000) Cloning and functional characterization of a 30 kb gene locus required for lipopolysaccharide biosynthesis in *Legionella pneumophila* . Int J Med Microbiol 290: 37–49. 11043980 10.1016/S1438-4221(00)80104-6

[67] Greenfield LK, Whitfield C (2012) Synthesis of lipopolysaccharide O-antigens by ABC transporter-dependent pathways. Carbohydr Res. 10.1016/j.carres.2012.02.027 22475157

[68] Whitfield C (2006) Biosynthesis and assembly of capsular polysaccharides in *Escherichia coli* . Annu Rev Biochem 75: 39–68. 16756484 10.1146/annurev.biochem.75.103004.142545

[69] Trent MS (2004) Biosynthesis, transport, and modification of lipid A. Biochem Cell Biol. 82: 71–86. 10.1139/o03-070 15052329

[70] Rangarajan ES, Proteau A, Cui Q, Logan SM, Potetinova Z, et al (2009) Structural and functional analysis of *Campylobacter jejuni* PseG: a udp-sugar hydrolase from the pseudaminic acid biosynthetic pathway. J Biol Chem 284: 20989–21000. 19483088 10.1074/jbc.M109.012351 PMC2742864

[71] Raymond CK, Sims EH, Kas A, Spencer DH, Kutyavin TV, et al (2002) Genetic variation at the O-antigen biosynthetic locus in *Pseudomonas aeruginosa* . J Bacteriol 184: 3614–3622. 12057956 10.1128/JB.184.13.3614-3622.2002 PMC135118

[72] Amaro F, Gilbert JA, Owens S, Trimble W, Shuman HA (2012) Whole-genome sequence of the human pathogen *Legionella pneumophila* serogroup 12 strain 570-CO-H. J Bacteriol 194: 1613–1614. 22374950 10.1128/JB.06626-11 PMC3294838

[73] Schroeder GN, Petty NK, Mousnier A, Harding CR, Vogrin AJ, et al (2010) *Legionella pneumophila* strain 130b possesses a unique combination of type IV secretion systems and novel Dot/Icm secretion system effector proteins. J Bacteriol 192: 6001–6016. 20833813 10.1128/JB.00778-10 PMC2976443

[74] Tsai CM, Frasch CE (1982) A sensitive silver stain for detecting lipopolysaccharides in polyacrylamide gels. Anal Biochem 119: 115–119. 6176137 10.1016/0003-2697(82)90673-x

[75] Ciesielski CA, Blaser MJ, Wang WL (1986) Serogroup specificity of *Legionella pneumophila* is related to lipopolysaccharide characteristics. Infect Immun 51: 397–404. 2417953 10.1128/iai.51.2.397-404.1986 PMC262339

[76] Thomason BM, Bibb WF (1984) Use of absorbed antisera for demonstration of antigenic variation among strains of *Legionella pneumophila* serogroup 1. J Clin Microbiol 19: 794–797. 6206087 10.1128/jcm.19.6.794-797.1984 PMC271187

[77] Joly JR, McKinney RM, Tobin JO, Bibb WF, Watkins ID, et al (1986) Development of a standardized subgrouping scheme for *Legionella pneumophila* serogroup 1 using monoclonal antibodies. J Clin Microbiol 23: 768–771. 3517064 10.1128/jcm.23.4.768-771.1986 PMC362834

[78] Pluschke G, Mayden J, Achtman M, Levine RP (1983) Role of the capsule and the O antigen in resistance of O18:K1 *Escherichia coli* to complement-mediated killing. Infect Immun 42: 907–913. 6196296 10.1128/iai.42.3.907-913.1983 PMC264385

[79] Grossman N, Schmetz MA, Foulds J, Klima EN, Jimenez-Lucho VE, et al (1987) Lipopolysaccharide size and distribution determine serum resistance in *Salmonella montevideo* . J Bacteriol 169: 856–863. 2433267 10.1128/jb.169.2.856-863.1987 PMC211858

[80] Goebel EM, Wolfe DN, Elder K, Stibitz S, Harvill ET (2008) O antigen protects *Bordetella parapertussis* from complement. Infect Immun 76: 1774–1780. 18285500 10.1128/IAI.01629-07 PMC2292887

[81] Schneider MC, Exley RM, Ram S, Sim RB, Tang CM (2007) Interactions between *Neisseria meningitidis* and the complement system. Trends Microbiol 15: 233–240. 17398100 10.1016/j.tim.2007.03.005

[82] Vogel U, Weinberger A, Frank R, Muller A, Kohl J, et al (1997) Complement factor C3 deposition and serum resistance in isogenic capsule and lipooligosaccharide sialic acid mutants of serogroup B *Neisseria meningitidis* . Infect Immun 65: 4022–4029. 9317002 10.1128/iai.65.10.4022-4029.1997 PMC175578

[83] Peitsch MC, Tschopp J (1991) Assembly of macromolecular pores by immune defense systems. Curr Opin Cell Biol 3: 710–716. 1722985 10.1016/0955-0674(91)90045-z

[84] Marcus RL, Shin HS, Mayer MM (1971) An alternate complement pathway: C-3 cleaving activity, not due to C4,2a, on endotoxic lipopolysaccharide after treatment with guinea pig serum; relation to properdin. Proc Natl Acad Sci U S A 68: 1351–1354. 5288385 10.1073/pnas.68.6.1351 PMC389187

[85] Daha MR (2010) Role of complement in innate immunity and infections. Crit Rev Immunol 30: 47–52. 20370619 10.1615/critrevimmunol.v30.i1.30

[86] Lachmann PJ, Thompson RA (1970) Reactive lysis: the complement-mediated lysis of unsensitized cells. II. The characterization of activated reactor as C56 and the participation of C8 and C9. J Exp Med 131: 643–657. 4193935 10.1084/jem.131.4.643 PMC2138770

[87] Rossi V, Wang Y, Esser AF (2010) Topology of the membrane-bound form of complement protein C9 probed by glycosylation mapping, anti-peptide antibody binding, and disulfide modification. Mol Immunol 47: 1553–1560. 20153530 10.1016/j.molimm.2010.01.013 PMC2849895

[88] Brieland J, Freeman P, Kunkel R, Chrisp C, Hurley M, et al (1994) Replicative *Legionella pneumophila* lung infection in intratracheally inoculated A/J mice. A murine model of human Legionnaires' disease. Am J Pathol 145: 1537–1546. 7992856 PMC1887509

[89] Ogawa M, Yoshida S, Mizuguchi Y (1994) 2-Deoxy-D-glucose inhibits intracellular multiplication and promotes intracellular killing of *Legionella pneumophila* in A/J mouse macrophages. Infect Immun 62: 266–270. 8262638 10.1128/iai.62.1.266-270.1994 PMC186096

[90] Takayanagi N, Ishiguro T, Matsushita A, Yoshii Y, Kagiyama N, et al (2009) Severe complications and their outcomes in 65 patients with *Legionella* pneumonia. Nihon Kokyuki Gakkai Zasshi 47: 558–568. 19637795

[91] Demello D, Kierol-Andrews L, Scalise PJ (2007) Severe sepsis and acute respiratory distress syndrome from community-acquired *Legionella* pneumonia: case report. Am J Crit Care 16: 320, 317. 17460326

[92] Pan YJ, Lin TL, Hsu CR, Wang JT (2011) Use of a *Dictyostelium* model for isolation of genetic loci associated with phagocytosis and virulence in *Klebsiella pneumoniae* . Infect Immun 79: 997–1006. 21173313 10.1128/IAI.00906-10 PMC3067516

[93] Touchon M, Hoede C, Tenaillon O, Barbe V, Baeriswyl S, et al (2009) Organised genome dynamics in the *Escherichia coli* species results in highly diverse adaptive paths. PLoS Genet 5: e1000344. 19165319 10.1371/journal.pgen.1000344 PMC2617782

[94] Canals R, Jimenez N, Vilches S, Regue M, Merino S, et al (2007) Role of Gne and GalE in the virulence of *Aeromonas hydrophila* serotype O34. J Bacteriol 189: 540–550. 17098903 10.1128/JB.01260-06 PMC1797372

[95] Kooistra O, Luneberg E, Lindner B, Knirel YA, Frosch M, et al (2001) Complex O-acetylation in *Legionella pneumophila* serogroup 1 lipopolysaccharide. Evidence for two genes involved in 8-O-acetylation of legionaminic acid. Biochemistry 40: 7630–7640. 11412117 10.1021/bi002946r

[96] Luck PC, Freier T, Steudel C, Knirel YA, Luneberg E, et al (2001) A point mutation in the active site of *Legionella pneumophila* O-acetyltransferase results in modified lipopolysaccharide but does not influence virulence. Int J Med Microbiol 291: 345–352. 11727818 10.1078/1438-4221-00140

[97] Zahringer U, Knirel YA, Lindner B, Helbig JH, Sonesson A, et al (1995) The lipopolysaccharide of *Legionella pneumophila* serogroup 1 (strain Philadelphia 1): chemical structure and biological significance. Prog Clin Biol Res 392: 113–139. 8524918

[98] Knirel YA, Senchenkova SN, Kocharova NA, Shashkov AS, Helbig JH, et al (2001) Identification of a homopolymer of 5-acetamidino-7-acetamido-3,5,7,9-tetradeoxy-D-glycero-D-talo-nonulosonic acid in the lipopolysaccharides of *Legionella pneumophila* Non-1 serogroups. Biochemistry (Mosc) 66: 1035–1041. 11703188 10.1023/a:1012334012605

[99] Otten S, Iyer S, Johnson W, Montgomery R (1986) Serospecific antigens of *Legionella pneumophila* . J Bacteriol 167: 893–904. 3017918 10.1128/jb.167.3.893-904.1986 PMC215957

[100] Lau PC, Lindhout T, Beveridge TJ, Dutcher JR, Lam JS (2009) Differential lipopolysaccharide core capping leads to quantitative and correlated modifications of mechanical and structural properties in *Pseudomonas aeruginosa* biofilms. J Bacteriol 191: 6618–6631. 19717596 10.1128/JB.00698-09 PMC2795305

[101] Wildschutte H, Wolfe DM, Tamewitz A, Lawrence JG (2004) Protozoan predation, diversifying selection, and the evolution of antigenic diversity in *Salmonella* . Proc Natl Acad Sci U S A 101: 10644–10649. 15247413 10.1073/pnas.0404028101 PMC489988

[102] Nakao R, Senpuku H, Watanabe H (2006) *Porphyromonas gingivalis gal*E is involved in lipopolysaccharide O-antigen synthesis and biofilm formation. Infect Immun 74: 6145–6153. 16954395 10.1128/IAI.00261-06 PMC1695533

[103] Holden EP, Winkler HH, Wood DO, Leinbach ED (1984) Intracellular growth of *Legionella pneumophila* within *Acanthamoeba castellanii* Neff. Infect Immun 45: 18–24. 6376354 10.1128/iai.45.1.18-24.1984 PMC263250

[104] Napoli C, Fasano F, Iatta R, Barbuti G, Cuna T, et al (2010) *Legionella spp*. and legionellosis in southeastern Italy: disease epidemiology and environmental surveillance in community and health care facilities. BMC Public Health 10: 660. 21044294 10.1186/1471-2458-10-660 PMC2988737

[105] Boccia S, Laurenti P, Borella P, Moscato U, Capalbo G, et al (2006) Prospective 3-year surveillance for nosocomial and environmental *Legionella pneumophila*: implications for infection control. Infect Control Hosp Epidemiol 27: 459–465. 16671026 10.1086/503642

[106] Harrison TG, Afshar B, Doshi N, Fry NK, Lee JV (2009) Distribution of *Legionella pneumophila* serogroups, monoclonal antibody subgroups and DNA sequence types in recent clinical and environmental isolates from England and Wales (2000–2008). Eur J Clin Microbiol Infect Dis 28: 781–791. 19156453 10.1007/s10096-009-0705-9

[107] Mekkour M, Ben-Driss EK, Cohen N (2012) Prevalence of *Legionella pneumophila* in production networks and distribution of domestic hot water in Morocco. World Environment 2: 11–15.

[108] Ewan BJDaP (1983) First Isolation of *Legionella pneumophila* from the Canadian Great Lakes. Journal of Great Lakes Research 9: 430–432.

[109] McCallum KL, Schoenhals G, Laakso D, Clarke B, Whitfield C (1989) A high-molecular-weight fraction of smooth lipopolysaccharide in *Klebsiella* serotype O1:K20 contains a unique O-antigen epitope and determines resistance to nonspecific serum killing. Infect Immun 57: 3816–3822. 2478478 10.1128/iai.57.12.3816-3822.1989 PMC259910

[110] Williams P, Lambert PA, Brown MR, Jones RJ (1983) The role of the O and K antigens in determining the resistance of *Klebsiella aerogenes* to serum killing and phagocytosis. J Gen Microbiol 129: 2181–2191. 6195306 10.1099/00221287-129-7-2181

[111] Mintz CS, Schultz DR, Arnold PI, Johnson W (1992) *Legionella pneumophila* lipopolysaccharide activates the classical complement pathway. Infect Immun 60: 2769–2776. 1612744 10.1128/iai.60.7.2769-2776.1992 PMC257233

[112] Kojouharova M, Reid K, Gadjeva M (2010) New insights into the molecular mechanisms of classical complement activation. Mol Immunol 47: 2154–2160. 20542571 10.1016/j.molimm.2010.05.011

[113] Conrad DH, Carlo JR, Ruddy S (1978) Interaction of beta1H globulin with cell-bound C3b: quantitative analysis of binding and influence of alternative pathway components on binding. J Exp Med 147: 1792–1805. 567241 10.1084/jem.147.6.1792 PMC2184316

[114] Bellinger-Kawahara C, Horwitz MA (1990) Complement component C3 fixes selectively to the major outer membrane protein (MOMP) of *Legionella pneumophila* and mediates phagocytosis of liposome-MOMP complexes by human monocytes. J Exp Med 172: 1201–1210. 2212949 10.1084/jem.172.4.1201 PMC2188623

[115] Ohno A, Isii Y, Tateda K, Matumoto T, Miyazaki S, et al (1995) Role of LPS length in clearance rate of bacteria from the bloodstream in mice. Microbiology 141 (Pt 10): 2749–2756. 10.1099/13500872-141-10-2749 7582035

[116] Plouffe JF, Para MF, Fuller KA (1985) Serum bactericidal activity against *Legionella pneumophila* . J Clin Microbiol 22: 863–864. 4056012 10.1128/jcm.22.5.863-864.1985 PMC268546

[117] Horwitz MA, Silverstein SC (1981) Interaction of the legionnaires' disease bacterium (*Legionella pneumophila*) with human phagocytes. II. Antibody promotes binding of *L. pneumophila* to monocytes but does not inhibit intracellular multiplication. J Exp Med 153: 398–406. 7241049 10.1084/jem.153.2.398 PMC2186075

[118] Ferguson JS, Weis JJ, Martin JL, Schlesinger LS (2004) Complement protein C3 binding to *Mycobacterium tuberculosis* is initiated by the classical pathway in human bronchoalveolar lavage fluid. Infect Immun 72: 2564–2573. 15102764 10.1128/IAI.72.5.2564-2573.2004 PMC387845

[119] Chiaraviglio L, Brown DA, Kirby JE (2008) Infection of cultured human endothelial cells by *Legionella pneumophila* . PLoS One 3: e2012. 18431493 10.1371/journal.pone.0002012 PMC2292252

[120] O'Connor TJ, Adepoju Y, Boyd D, Isberg RR (2011) Minimization of the Legionella pneumophila genome reveals chromosomal regions involved in host range expansion. Proc Natl Acad Sci U S A 108: 14733–14740. 21873199 10.1073/pnas.1111678108 PMC3169125

[121] Cherry WB, Pittman B, Harris PP, Hebert GA, Thomason BM, et al (1978) Detection of Legionnaires disease bacteria by direct immunofluorescent staining. J Clin Microbiol 8: 329–338. 359594 10.1128/jcm.8.3.329-338.1978 PMC275241

[122] Thacker WL, Wilkinson HW, Benson RF (1983) Comparison of slide agglutination test and direct immunofluorescence assay for identification of Legionella isolates. J Clin Microbiol 18: 1113–1118. 6358251 10.1128/jcm.18.5.1113-1118.1983 PMC272851

[123] Tijet N, Tang P, Romilowych M, Duncan C, Ng V, et al (2010) New endemic *Legionella pneumophila* serogroup I clones, Ontario, Canada. Emerg Infect Dis 16: 447–454. 20202420 10.3201/eid1603.081689 PMC3322000

[124] Duncan C, Prashar A, So J, Tang P, Low DE, et al (2011) Lcl of *Legionella pneumophila* is an immunogenic GAG binding adhesin that promotes interactions with lung epithelial cells and plays a crucial role in biofilm formation. Infect Immun 79: 2168–2181. 21422183 10.1128/IAI.01304-10 PMC3125840

[125] Lebeau I, Lammertyn E, De Buck E, Maes L, Geukens N, et al (2004) Novel transcriptional regulators of *Legionella pneumophila* that affect replication in *Acanthamoeba castellanii* . Arch Microbiol 181: 362–370. 15034642 10.1007/s00203-004-0664-6

[126] Ensminger AW, Yassin Y, Miron A, Isberg RR (2012) Experimental Evolution of *Legionella pneumophila* in Mouse Macrophages Leads to Strains with Altered Determinants of Environmental Survival. PLoS Pathog 8: e1002731. 22693450 10.1371/journal.ppat.1002731 PMC3364954

[127] Meyer F, Goesmann A, McHardy AC, Bartels D, Bekel T, et al (2003) GenDB–an open source genome annotation system for prokaryote genomes. Nucleic Acids Res 31: 2187–2195. 12682369 10.1093/nar/gkg312 PMC153740

[128] Reimer AR, Van Domselaar G, Stroika S, Walker M, Kent H, et al (2011) Comparative genomics of *Vibrio cholerae* from Haiti, Asia, and Africa. Emerg Infect Dis 17: 2113–2121. 22099115 10.3201/eid1711.110794 PMC3310578

[129] Linke B, McHardy AC, Neuweger H, Krause L, Meyer F (2006) REGANOR: a gene prediction server for prokaryotic genomes and a database of high quality gene predictions for prokaryotes. Appl Bioinformatics 5: 193–198. 16922601 10.2165/00822942-200605030-00008

[130] Badger JH, Olsen GJ (1999) CRITICA: coding region identification tool invoking comparative analysis. Mol Biol Evol 16: 512–524. 10331277 10.1093/oxfordjournals.molbev.a026133

[131] Delcher AL, Bratke KA, Powers EC, Salzberg SL (2007) Identifying bacterial genes and endosymbiont DNA with Glimmer. Bioinformatics 23: 673–679. 17237039 10.1093/bioinformatics/btm009 PMC2387122

[132] Suzek BE, Ermolaeva MD, Schreiber M, Salzberg SL (2001) A probabilistic method for identifying start codons in bacterial genomes. Bioinformatics 17: 1123–1130. 11751220 10.1093/bioinformatics/17.12.1123

[133] Schattner P, Brooks AN, Lowe TM (2005) The tRNAscan-SE, snoscan and snoGPS web servers for the detection of tRNAs and snoRNAs. Nucleic Acids Res 33: W686–689. 15980563 10.1093/nar/gki366 PMC1160127

[134] Lagesen K, Hallin P, Rodland EA, Staerfeldt HH, Rognes T, et al (2007) RNAmmer: consistent and rapid annotation of ribosomal RNA genes. Nucleic Acids Res 35: 3100–3108. 17452365 10.1093/nar/gkm160 PMC1888812

[135] Altschul SF, Madden TL, Schaffer AA, Zhang J, Zhang Z, et al (1997) Gapped BLAST and PSI-BLAST: a new generation of protein database search programs. Nucleic Acids Res 25: 3389–3402. 9254694 10.1093/nar/25.17.3389 PMC146917

[136] Rice P, Longden I, Bleasby A (2000) EMBOSS: the European Molecular Biology Open Software Suite. Trends Genet 16: 276–277. 10827456 10.1016/s0168-9525(00)02024-2

[137] Finn RD, Mistry J, Tate J, Coggill P, Heger A, et al (2010) The Pfam protein families database. Nucleic Acids Res 38: D211–222. 19920124 10.1093/nar/gkp985 PMC2808889

[138] Haft DH, Selengut JD, White O (2003) The TIGRFAMs database of protein families. Nucleic Acids Res 31: 371–373. 12520025 10.1093/nar/gkg128 PMC165575

[139] Kanehisa M, Goto S, Sato Y, Furumichi M, Tanabe M (2012) KEGG for integration and interpretation of large-scale molecular data sets. Nucleic Acids Res 40: D109–114. 22080510 10.1093/nar/gkr988 PMC3245020

[140] Sonnhammer EL, von Heijne G, Krogh A (1998) A hidden Markov model for predicting transmembrane helices in protein sequences. Proc Int Conf Intell Syst Mol Biol 6: 175–182. 9783223

[141] Nielsen H, Brunak S, von Heijne G (1999) Machine learning approaches for the prediction of signal peptides and other protein sorting signals. Protein Eng 12: 3–9. 10065704 10.1093/protein/12.1.3

[142] Petkau A, Stuart-Edwards M, Stothard P, Van Domselaar G (2010) Interactive microbial genome visualization with GView. Bioinformatics 26: 3125–3126. 20956244 10.1093/bioinformatics/btq588 PMC2995121

[143] McKinney RM, Thomason BM, Harris PP, Thacker L, Lewallen KR, et al (1979) Recognition of a new serogroup of Legionnaires disease bacterium. J Clin Microbiol 9: 103–107. 372210 10.1128/jcm.9.1.103-107.1979 PMC272965

