# Supplementary material for: Comparative Genomics Reveal That Host-Innate Immune Responses Influence the Clinical Prevalence of Legionella pneumophila Serogroups
Source: PLoS One. 2013 Jun 27;8(6):e67298. doi: 10.1371/journal.pone.0067298 (PMC3694923; doi:10.1371/journal.pone.0067298)
Supplement: Figure S2 — MLVA typing of a population based clinical repository of L. pneumophila . Phylogenetic clusters were constructed based on UPGMA analysis of MLVA based distribution. The large cluster and the subclusters 1 and 2 are identified in the phylogenetic tree. (PDF) [file pone.0067298.s002.pdf]

Figure S2

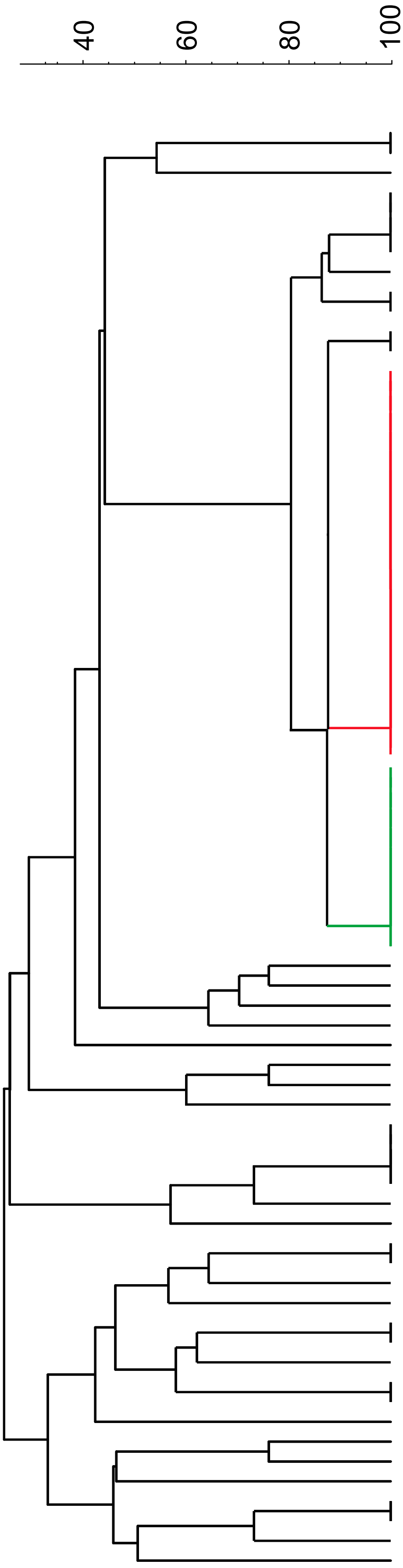

MLVA Markers									Molecular types		Serogroups	
Sg13	Sg35	Sg33	Sg34	Sg31	Sg17	Sg19	Sg01	Sg03	MLVA	ST		
3.0	8.0	2.5	1.0	16.0			8.0	8.0	is310	471	Sg 5	
3.0	8.0	2.5	1.0		2.0	4.0	8.0	8.0	is386	·	Sg5	
3.0	11.0	1.0	1.0	16.0		4.0	7.5	8.0	is332	·	Sg4	
11.0	3.0	1.0	1.0	17.0	2.0	4.0	8.0	8.0	is316	114	Sg6	
11.0	3.0	1.0	1.0	17.0	2.0	4.0	8.0	8.0	is330	621	Sg5	
11.0	3.0	1.0	1.0	17.0	2.0	4.0	8.0	8.0	is359	114	Sg6	
11.0	3.0	1.0	1.0	17.0	2.0	4.0	8.0	8.0	is376	·	Sg5	
9.0	3.0	1.0	1.0	17.0	2.0	4.0	8.0	8.0	is321	187	Sg6	
10.0	3.0	1.0	1.0	17.0	2.0		8.0	8.0	is339	187	Sg6	
10.0	3.0	1.0	1.0		2.0	4.0	8.0	8.0	is388	·	Sg8	
14.0	3.0	1.0	1.0	17.0	1.5	4.0	8.0	8.0	is365	187	Sg6	
14.0	3.0	1.0	1.0	17.0	1.5	4.0	8.0	8.0	is372	187	Sg6	
11.0	3.0	1.0	1.0	17.0	1.5		8.0	8.0	is315	187	Sg6	(THUNDER BAY)
11.0	3.0	1.0	1.0	17.0	1.5	4.0	8.0	8.0	is317	68	Sg6	(SUDBURY)
11.0	3.0	1.0	1.0	17.0	1.5	4.0	8.0	8.0	is324	68	Sg6	
11.0	3.0	1.0	1.0	17.0	1.5	4.0	8.0	8.0	is333	469	Sg10	
11.0	3.0	1.0	1.0	17.0	1.5	4.0	8.0	8.0	is337	68	Sg6	
11.0	3.0	1.0	1.0	17.0	1.5	4.0	8.0	8.0	is344	187	Sg6	
11.0	3.0	1.0	1.0	17.0	1.5	4.0	8.0		is345	468	Sg8	
11.0	3.0	1.0	1.0	17.0	1.5	4.0	8.0	8.0	is346	68	Sg6	
11.0	3.0	1.0	1.0	17.0	1.5	4.0	8.0	8.0	is347	68	Sg6	
11.0	3.0	1.0	1.0	17.0	1.5	4.0	8.0	8.0	is348	68	Sg6	
11.0	3.0	1.0	1.0	17.0	1.5	4.0	8.0	8.0	is349	378	Sg4	
11.0	3.0	1.0	1.0	17.0	1.5	4.0	8.0	8.0	is350	187	Sg6	
11.0	3.0	1.0	1.0	17.0	1.5	4.0	8.0	8.0	is352	187	Sg6	
11.0	3.0	1.0	1.0	17.0	1.5		8.0	8.0	is363	68	Sg6	
11.0	3.0	1.0	1.0	17.0	1.5	4.0	8.0	8.0	is364	68	Sg6	
11.0	3.0	1.0	1.0	17.0	1.5	4.0	8.0	8.0	is366	187	Sg8	
11.0	3.0	1.0	1.0	17.0	1.5	4.0	8.0	8.0	is367	·	Sg8	
11.0	3.0	1.0	1.0	17.0	1.5	4.0	8.0	8.0	is368	·	Sg4-8	
11.0	3.0	1.0	1.0	17.0	1.5	4.0	8.0	8.0	is374	68	Sg6	
11.0	3.0	1.0	1.0	17.0	1.5	4.0	8.0	8.0	is377	378	Sg4	
10.0	3.0	1.0	1.0	17.0	1.5	4.0	8.0	8.0	is309	242	Sg6	
10.0	3.0	1.0	1.0	17.0	1.5	4.0	8.0	8.0	is341	187	Sg6	
10.0	3.0	1.0	1.0	17.0	1.5	4.0	8.0	8.0	is353	187	Sg6	
10.0	3.0	1.0	1.0	17.0	1.5	4.0	8.0	8.0	is355	187	Sg6	
10.0	3.0	1.0	1.0	17.0	1.5	4.0	8.0	8.0	is369	187	Sg6	
10.0	3.0	1.0	1.0	17.0	1.5	4.0	8.0	8.0	is371	187	Sg6	
10.0	3.0	1.0	1.0	17.0	1.5	4.0	8.0	8.0	is373	93	Sg3	
10.0	3.0	1.0	1.0	17.0	1.5		8.0	8.0	is379	187	Sg6	
10.0	3.0	1.0	1.0	17.0	1.5		8.0	8.0	is380	187	Sg6	
10.0	3.0	1.0	1.0	17.0	1.5	4.0	8.0	8.0	is381	187	Sg6	
10.0	13.0	4.0	1.0	15.0	2.0	5.0	8.0	8.0	is319	470	Sg10	
10.0	12.0	4.0	1.0	17.0	2.0	5.0	8.0	8.0	is360	395	Sg3	
10.0	3.0	4.0	1.0	17.0	2.0	4.0	8.0	8.0	is357	467	Sg5	
10.0	13.0	4.0	1.0	14.0	2.0	5.0	7.0	8.0	is362	60	Sg6	
7.0	14.0	3.0	1.0	15.0	1.5	5.0	8.0	8.0	is378	460	Sg5	
12.0	6.0	3.0	2.0	16.0	1.5	4.0	7.0	8.0	is328	39	Sg2	
8.0	3.0	3.0	2.0	16.0	1.5	4.0	7.0	8.0	is343	39	Sg2	
16.0	14.0	3.0	2.0		2.0	4.0	7.0	8.0	is383	226	Sg8	
12.0	16.0	4.0	1.0	19.0	2.0	4.0	7.5	7.0	is313	·	Sg4	
12.0	16.0	4.0	1.0	19.0	2.0	4.0	7.5	7.0	is320	·	Sg8	
12.0	16.0	4.0	1.0		2.0	4.0	7.5	7.0	is338	·	Sg8	
12.0	16.0	4.0	1.0		2.0	4.0	7.5	7.0	is384	·	Sg8	
12.0	18.0	1.0	1.0		2.0	4.0	7.5	7.0	is382	·	Sg8	
10.0	15.0	3.0	1.0	13.0	2.0	4.0	7.5	7.0	is340	·	Sg4	
8.0	24.0	2.0	3.0	14.0	2.0	4.0	9.0	8.0	is336	·	Sg4	
8.0	24.0	2.0	3.0	14.0	2.0	4.0	9.0	8.0	is370	466	Sg8	
10.0	21.0	2.0	3.0	14.0	2.0	4.0	8.0	8.0	is307	472	Sg8	
8.0	11.0	2.0	3.0	10.0	2.0	5.0	9.0	8.0	is308	473	Sg12	
10.0	18.0	4.0	2.0	14.0	2.0	4.0	9.0	8.0	is311	407	Sg6	
10.0	18.0	4.0	2.0	14.0		4.0	9.0	8.0	is331	407	Sg6	(MISSISSAUGA)
8.0	18.0	2.0	2.0	13.5	2.0	4.0	9.0	8.0	is351	367	Sg6	
7.0	18.0	2.0	2.0	14.0	2.0	5.0		8.0	is318	464	Sg3	
7.0	18.0	2.0	2.0	14.0	2.0		7.0	8.0	is361	464	Sg2-3	
11.0	17.0	3.0	4.0	14.0	2.0	4.0	9.0	8.0	is342	461	Sg6	
7.0	28.0	2.0	3.0	15.0	2.0	4.0	8.0	7.0	is322	463	Sg10	
7.0	23.0	2.0	3.0	14.5	2.0	4.0	8.0	7.0	is323	·	Sg10	
4.0	26.0	2.0	1.0	17.0	2.0		8.0	7.0	is358	462	Sg3	
4.0	23.0	2.0	4.0	15.0	2.0	4.0	8.0	8.0	is314	465	Sg3	
4.0	23.0	2.0	4.0	15.0	2.0	4.0	8.0	8.0	is356	465	Sg3	
4.0	23.0	2.0	2.0		2.0	4.0	7.0	8.0	is387	710	Sg5	
9.0	19.0	2.0	1.0	10.0	2.0	4.0	8.0	8.0	is312		Sg8	

Subcluster 1 (is315–is377)

Subcluster 2 (is309–is381)

Large cluster (is316–is381)
