# Supplementary material for: Comparative Genomics Reveal That Host-Innate Immune Responses Influence the Clinical Prevalence of Legionella pneumophila Serogroups
Source: PLoS One. 2013 Jun 27;8(6):e67298. doi: 10.1371/journal.pone.0067298 (PMC3694923; doi:10.1371/journal.pone.0067298)
Supplement: Table S1 — General features of the sequenced Sg6 strains. (PDF) [file pone.0067298.s003.pdf]

Table S1:

	Thunder Bay	Sudbury	Mississauga
**Genome Size (b.p.)**	3,455,167	3,352,937	3,385,633
**% GC**	38.23	38.19	38.19
**CDS**	3089	2898	2884
**Coverage**	118X	98X	98X
**Number of SNP with Thunder Bay**	-	4,817	79,039
**% of SNPs in CDS**	-	88.18	63.62
