# Supplementary material for: Comparative Genomics Reveal That Host-Innate Immune Responses Influence the Clinical Prevalence of Legionella pneumophila Serogroups
Source: PLoS One. 2013 Jun 27;8(6):e67298. doi: 10.1371/journal.pone.0067298 (PMC3694923; doi:10.1371/journal.pone.0067298)
Supplement: Table S2 — Comparative proteomics analysis between Sg1 core genes and Sg6 str. Thunder Bay. Proteins that share less than 65% sequence homology and 75% coverage were defined as divergent. HP identifies hypothetical proteins. (DOC) [file pone.0067298.s004.doc]

**Table S2. Comparative proteomics analysis between Sg1 core genes and Sg6 str. Thunder Bay.** Proteins that share less than 65% sequence homology and 75% coverage were defined as divergent. HP identifies hypothetical proteins.

| **Sg1 Core** | **Sg1 Gene** | **Gene Description** | **Identity to Sg6** | **Sg6 Gene** | **Locus Tag** | **Sg6 Gene Description** |
| --- | --- | --- | --- | --- | --- | --- |
| lpg0031 |  | HP | 0 |  |  |  |
| lpg0190 |  | HP | 37 | TrmE | lp6_2961 | DnaA-Like |
| lpg0193 |  | HP | 44 |  | lp6_2206 | Isovaleryl CoA Dehydrogenase |
| lpg0216 |  | HP | 39 |  | lp6_1097 | Sensory box protein |
| lpg0305 |  | HP | 41 | FimT | lp6_631 | Type IV-pre pillin |
| lpg0333 |  | HP | 32 |  | lp6_1147 | HP |
| lpg0398 |  | HP | 0 |  |  |  |
| lpg0465 |  | HP | 45 |  | lp6_57 | Hydrolase |
| lpg0509 |  | Sugar transporter family | 42 |  | lp6_1849 | Transglutaminase domain-containing |
| lpg0523 |  |  | 52 | Cyc5 | lp6_908 | Cytochrome C5 |
| lpg0538 |  | HP | 53 |  | lp6_558 | Ribose-phosphate pyrophosphokinase |
| lpg0566 | SmpB | SsrA-binding protein | 61 |  | lp6_2918 | Stomatin like transmembrane protein |
| lpg0567 |  | Peptidase | 37 |  | lp6_74 | Peptidase |
| lpg0572 |  | HP | 41 | FimT | lp6_631 | Type IV-pre pillin |
| lpg0606 |  | Metal-sulfur enzyme | 43 | GuaA | lp6_1681 | GMP synthase |
| lpg0668 |  | HP | 0 |  |  |  |
| lpg0705 |  | transporter | 0 |  |  |  |
| lpg0743 |  | Glutamate Synthase | 40 |  | lp6_2776 | Tyrosine phosphatase II superfamily protein |
| lpg0761 | GalE | UDP-galactose-4-epimerase | 60 | GalE | lp6_758 | UDP-galactose-4-epimerase |
| lpg0762 | SecA | α-*N*-acetylglucose-aminyltransferase | 55 | WecA | lp6_759 | α-*N*-acetylglucose-aminyltransferase |
| lpg0766 |  | HP | 48 |  | lp6_751 | HP |
| lpg0768 | NeuB | *N*-acetylneuraminic acid synthetase | 35 | NeuB | lp6_745 | *N*-acetylneuraminic acid synthetase |
| lpg0769 |  | HP | 50 | RsmB | lp6_2562 | Ribosomal RNA small subunit methyltransferase |
| lpg0770 |  | HP | 36 | VipE | lp6_2769 | Unknown function |
| lpg0772 | Wzm | LPS O-antigen ABC transporter | 46 | Wzm | lp6_749 | LPS O-antigen ABC transporter |
| lpg0773 | Wzt | LPS O-antigen ABC transporter | 63 | Wzt | lp6_750 | LPS O-antigen ABC transporter |
| lpg0774 |  | HP | 50 |  | lp6_755 | HP |
| lpg0777 | Lag1 | *O*-acetyl transferase | 34 | AlgL | lp6_760 | Alginate O-acetyl transferase |
| lpg0796 |  | HP | 36 | SidF | lp6_2553 | Inhibitor of growth protein |
| lpg0813 | MreD | O-acetyltransferase | 35 | RpoH | lp6_2636 | RNA polymerase factor σ_32_ |
| lpg0820 |  | Endonuclease | 58 |  | lp6_506 | Endonuclease |
| lpg0823 |  | Neurogenic locus protein | 40 | DsbD1 | lp6_683 | Thiol:disulfide interchange protein |
| lpg0913 | MraZ |  | 50 | HelB | lp6_1022 | Cation efflux system |
| lpg1045 |  | HP | 26 | GyrA2 | lp6_1392 | DNA gyrase subunit A |
| lpg1046 |  | HP | 39 | RpsD | lp6_375 | 30S ribosomal protein S4 |
| lpg1120 |  | HP | 50 | Eno | lp6_1985 | Phosphopyruvate hydratase |
| lpg1133 |  | HP | 45 | LvhB11 | lp6_1228 | *Legionella* vir homologue protein |
| lpg1258 | LvrB | *Legionella* vir region protein | 30 | YggT | lp6_1965 | Osmotic regulation |
| lpg1261 |  | HP | 38 | FolC | lp6_1317 | Bifunctional folylpolyglutamate synthase/dihydrofolate synthase |
| lpg1476 |  | HP | 53 |  | lp6_287 | Acetyltransferase |
| lpg1505 |  | HP | 54 |  | lp6_1381 | Glycosyltransferase |
| lpg1578 |  | HP | 48 | SulP | lp6_2156 | Sulfate permease |
| lpg1802 |  | HP | 39 | YbdK | lp6_614 | Carboxylate amine ligase |
